# Continuous Flow Oxidation of Alcohols Using TEMPO/NaOCl for the Selective and Scalable Synthesis of Aldehydes

**DOI:** 10.1021/acs.oprd.3c00237

**Published:** 2023-09-07

**Authors:** Parth Naik, Jorge García-Lacuna, Patrick O’Neill, Marcus Baumann

**Affiliations:** † School of Chemistry, University College Dublin, Science Centre South, Belfield D04 N2E5, Ireland; ‡ Pfizer Ireland, Ringaskiddy P43 X336, Ireland

**Keywords:** TEMPO-oxidation, continuous flow, in-line extraction, aldehyde, scale-up

## Abstract

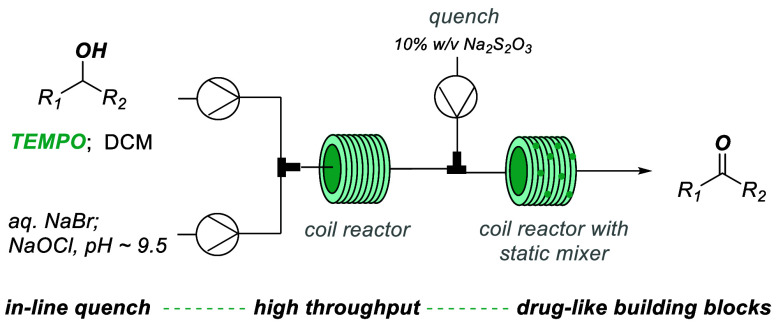

A simple and benign continuous flow oxidation protocol for the selective conversion of primary and secondary alcohols into their respective aldehyde and ketone products is reported. This approach makes use of catalytic amounts of TEMPO in combination with sodium bromide and sodium hypochlorite in a biphasic solvent system. A variety of substrates are tolerated including those containing heterocycles based on potentially sensitive nitrogen and sulfur moieties. The flow approach can be coupled with inline reactive extraction by formation of the carbonyl-bisulfite adduct which aids in separation of remaining substrate or other impurities. Process robustness is evaluated for the preparation of phenylpropanal at decagram scale, a trifluoromethylated oxazole building block as well as a late-stage intermediate for the anti-HIV drug maraviroc which demonstrates the potential value of this continuous oxidation method.

## Introduction

Oxidation reactions remain a main staple in the chemist’s repertoire of important synthetic transformations. Of particular interest are thereby oxidations that use readily affordable and nontoxic oxidants that are often accompanied by simple yet robust catalysts. Among such catalyzed oxidation processes, the use of TEMPO (2,2,6,6-tetramethylpiperidin-1-yl)oxyl) is enjoying popularity in both industrial and academic applications for the selective oxidation of alcohols to aldehydes and ketones.^1^ Recent studies by Stahl^2^ and co-workers showcase the value of this methodology in Cu-catalyzed oxidations using oxygen as a stoichiometric oxidant. A related system that is often employed in industry uses TEMPO in combination with bleach (NaOCl) and sodium bromide as cocatalyst^1,3^ or other variants include the use of 4-hydroxy-TEMPO.^4^

Continuous flow processing^5^ has been exploited in many recent applications to render oxidation reactions safe irrespective of the reaction scale.^6^ This is due to improved heat transfer in miniaturized set-ups that facilitates dissipation of heat from these exothermic processes. Additional benefits of flow over batch processing such as better mass transfer, intrinsic scalability, reaction telescoping as well as integrated analysis and reaction quenching are commonly cited and account for the high popularity of continuous flow processes.^7^ Unsurprisingly, a number of oxidative transformations using flow processing have been reported over recent years including the use of bleach in combination with catalytic TBAB (tetrabutylammonium bromide),^8^ the use of potassium permanganate in Nef oxidations,^9^ aerobic oxidations using transition metal catalysts,^6a,10^ biocatalyzed oxidations,^11^ electrochemical oxidations,^12^ and multiple examples exploiting the in situ generation of harmful oxidants such as ozone,^13^ performic acid,^14^ and others.^6^

We set out to establish a continuous flow oxidation protocol for alcohols using TEMPO in combination with bleach as a benign and affordable oxidant combination. This was fuelled by our desire to develop a metal-free process characterized by high throughput and easy product isolation. In addition, we wished to realize a robust process that would be selective for the generation of aldehydes from primary alcohols while tolerating a variety of additional functionalities including various heterocyclic motifs as commonly encountered in drugs and their building blocks.

## Results and Discussion

**Scheme 1:**
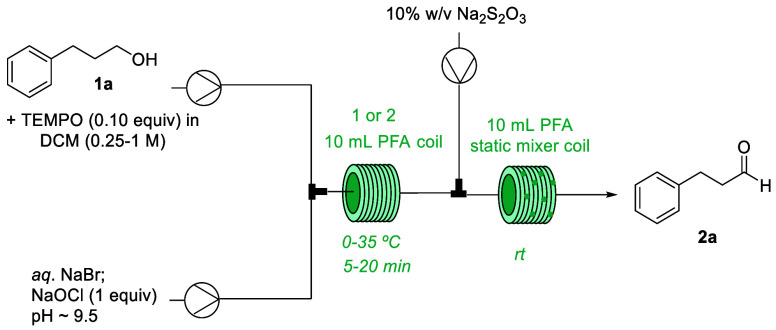
General Set-up for Reaction Optimization of Substrate **1a**

Inspired by recent reports of TEMPO-catalyzed oxidation reactions in batch mode we commenced our investigation by performing optimization studies evaluating the influence of several parameters (residence time, reactor volume, temperature) using 3-phenylpropanol (**1a**) as the model substrate. This compound was chosen as reported batch protocols indicated the potential for high conversion (up to 90%) and selectivity toward the corresponding aldehyde.^15^ However, competitive overoxidation forming the corresponding acid side-product further encouraged us to study the specific effect of reactor volume, residence time, and temperature to achieve process robustness and selectivity. The classical Anelli-Montanari protocol tends to use high concentrations of alcohol substrates in DCM (2 M),^3^ however, for our lab scale settings the concentration was decreased to 0.25–1 M in DCM, which translated into equal volumes for organic and aqueous solutions. This was an advantage when transferring batch conditions to flow mode to achieve uniform plug flow. In our case, the initial batch experiment with these conditions provided 3-phenylpropanal **2a** in a 90% yield (via quantitative ^1^H NMR) after 1 h reaction time at ambient temperature. Next, we initiated our flow studies with the setup as depicted in Scheme 1. After testing different types of pumps (peristaltic, syringe, and piston pumps), peristaltic pumps were chosen as they offered consistent performance at steady state and better results (see Supporting Information, SI, for more information). In addition, for a lab scale settings syringe pumps might not be suitable for multigram scale-ups due to limited syringe volumes, whereas piston pumps are often less desirable for volatile solvent such as DCM.

Table 1 shows the optimization study varying the residence time at room temperature (ca. 20 °C). It was found that a yield of 86% (by ^1^H NMR) was obtained for a residence time of 7.5 min (entry 2) which was a slight improvement over shorter or longer residence times (entries 1 and 3). A slight increase in yield for **2a** up to 90% was achieved when the flow rate was doubled while keeping the residence time constant which can be attributed to faster flow rates and the associated increase in mixing, which demonstrates the beneficial effect of turbulence for biphasic systems (entry 6).^16^ Another feed was then included delivering a solution of Na_2_S_2_O_3_ to quench the reaction mixture inline followed by an internal static mixer coil (10 mL, PFA) to enhance mixing of the system. This inline quench is important to avoid nitroxyl radicals in the crude mixture as they present genotoxic activity which needs to be avoided in pharmaceutical manufacturing.^17^ Next, the effect of varying temperature and concentration was studied. The reaction rate tends to decrease with an increase in temperature as oxoammonium salts (i.e., TEMPO) are unstable and decompose rapidly at temperatures above 25 °C.^18^ Therefore, to find the optimum temperature window experiments were performed over a range of temperatures using either air or water as a heat transfer medium (see SI for details). Use of a water bath to control the coil temperature can prevent localized exotherms that might affect the yield of a reaction. It was evident that when reactions were performed at the same temperature with vs without a water bath there was a slight difference in yields particularly at 20–22 °C which suggests better heat transfer (better conduction when using a water bath) helps in achieving better control over the reaction exotherm (entries 7 and 8). The study further suggests that for flow conditions the optimum temperature lies between 10–20 °C. It should be noted that yields were higher in the absence of a water bath, however, to quench the exothermicity of the reaction and achieve better heat transfer a water bath was preferred for substrate scope and future reactions. The possibility of performing the reaction at room temperature in flow, instead of 0 °C like the typical batch procedure, was also observed by Pagliaro and co-workers in their heterogeneous protocol without KBr.^19^ Finally, entry 9 shows the importance of using a fresh solution of NaOCl as the yield dropped to 60% when using a less concentrated solution of aged NaOCl (0.26 M).

**Table 1:** Optimization Studies for Model Substrate **1a**

entry^a^	residence time (min)	flow rate (mL/min)	reactor vol. (mL)	NMR yield^b^ (%)
**1**	5	1.00	10	84
**2**	7.5	0.67	10	86
**3**	10	0.50	10	82
**4**	15	0.34	10	68
**5**	20	0.25	10	60
**6**	7.5	1.34	20	90
**7**^c^	7.5	1.34	20	93
**8**^d^	7.5	1.34	20	86
**9**^e^	7.5	1.34	20	60

a General conditions: Using two peristaltic pumps (flow rate of each pump) *T* = 17 °C, Feed 1: conc. [NaOCl] = 0.34 M, and NaBr (0.23 equiv) TEMPO equiv: 0.10, feed 2: alcohol **1a** in DCM (0.25 M).

b Calculated by qNMR using 1,3,5-trimethoxybenzene was used as an internal standard.

c Reaction performed at 23 °C using air as heat transfer medium and [NaOCl] = 2.21 M, alcohol **1a** in DCM (1 M).

d Reaction performed at 23 °C using a water bath as heat transfer medium with the conditions of entry 7.

e Using a 0.26 M [NaOCl] solution with a 0.25 M concentration of alcohol.

**Scheme 2:**
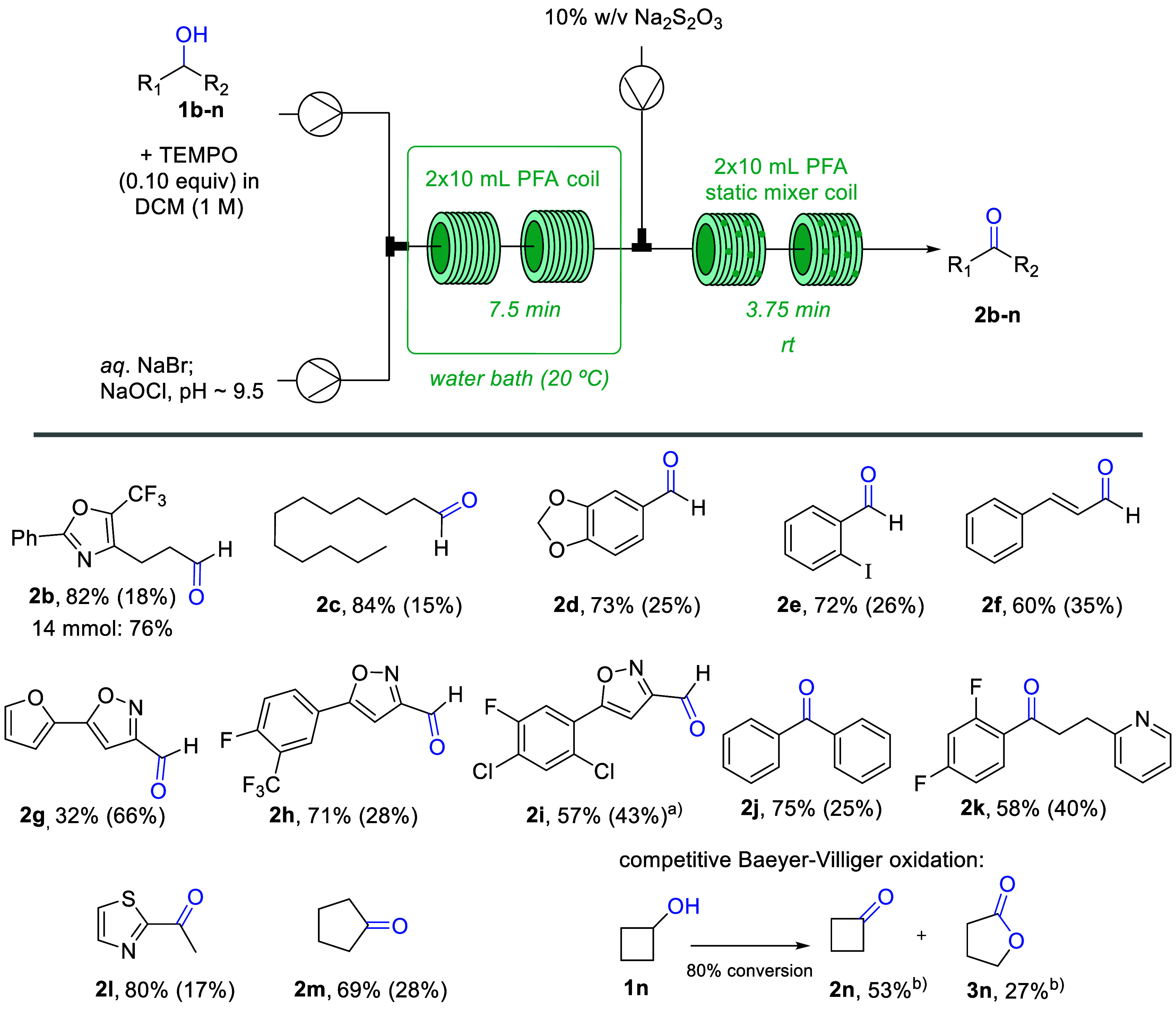
Scope of Primary and Secondary Alcohols in the Flow Oxidation Unless otherwise noted, yields are isolated yields after column chromatography, numbers in brackets indicate amount of unreacted starting material observed in the crude; 0.73-1 mmol scale, ^a)^ substrate 0.25 M (DCM) and 0.32 M NaOCl (aq). ^b)^ qNMR yield using 1,3,5-trimethoxybenzene as internal standard.

To demonstrate the scope for this transformation a selection of primary and secondary alcohols was studied using the optimized reaction conditions (Scheme 2). The developed flow method worked well for producing a variety of aldehyde and ketone products. Several examples of aliphatic (**2b**–**2c**) and benzylic alcohols (**2d**–**2e**) were oxidized in good to excellent yields, including the oxidation of primary alcohols present in heterocycles such as oxazoles (**2b**) and isoxazoles (**2g**–**2i**). No overoxidation was observed in any of these cases. Furthermore, secondary alcohols were also cleanly oxidized to the corresponding ketone products in good to excellent yields (**2j**–**2m**). The presence of heterocyclic systems that could undergo oxidation, such as pyridines (**2k**) or thiazoles (**2l**), was thereby well tolerated.

Interestingly, cyclobutanol (**1n**) produced the anticipated cyclobutanone product **2n** in only 53%, along with lactone **3n** in 27% yield. No lactone formation was observed during the oxidation of the related cyclopentanol **1m**. There are a few reports for the oxidation of cyclobutanol with concomitant formation of this γ-lactone under various oxidative conditions,^20^ however, little is known about the Baeyer–Villiger oxidation of cyclic ketones to lactones in the presence of HOCl at different pH (4–12).^21^ Hypochlorous acid resembles peroxyacids being both a weak acid and an oxidizing agent. The driving force is the release of ring strain which accounts for the formation of the lactone product in case of cyclobutanol. To suppress lactone formation, experiments at lower temperature and shorter residence time were performed, however, at 10 °C low substrate conversion was observed and the lactone remained the major product (see SI for more information). Ultimately, a maximum conversion of starting material of 80% was achieved following the general procedure indicating that lactone **3n** forms rapidly during the oxidation of **1n** to **2n**.

### Telescoped Continuous Extractive Workup of Cyclobutanone

**Scheme 3:**
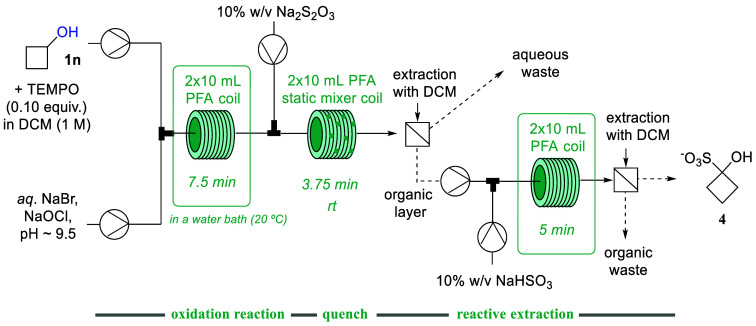
Telescoped Continuous Synthesis and Extractive Workup of Cyclobutanone

As the synthesis of cyclobutanone from cyclobutanol represented an interesting case whereby a side-product (i.e., lactone **3n**) was invariably generated, we wished to evaluate whether the desired cyclobutanone product could be separated from the lactone via a continuous extraction process. To achieve this a stream containing aqueous NaHSO_3_ (10% w/v) was mixed with the organic phase containing the crude reaction mixture to form the cyclobutanone-bisulfite adduct which is water-soluble and thus can be separated easily from the reaction mixture (Scheme 3). The resulting biphasic mixture passed through two additional tubular coils (2 × 10 mL, PFA, 10 min *t*_Res_ combined) prior to gravity based phase separation. ^1^H NMR analysis of the crude mixture before and after extraction with NaHSO_3_ showed that 74% of the available cyclobutanone was extracted from the crude mixture representing an overall yield of 39%.

**Figure 1:**
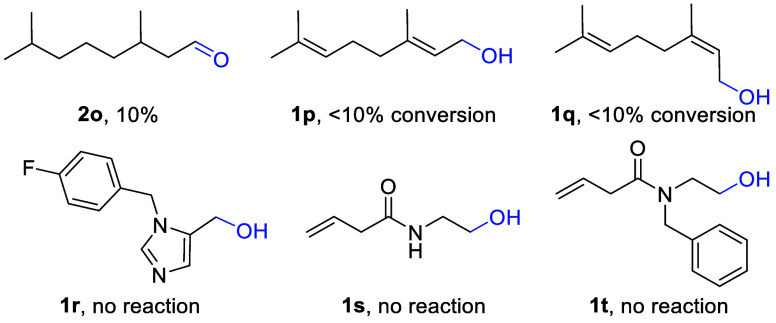
Unsuccessful substrates in flow Anelli-Montanari oxidation.

Despite the high success rate of this flow-based TEMPO oxidation protocol, not all the substrates explored gave high yields and some showed inhibited oxidation (Figure 1), thus indicating that alternative oxidation protocols would be needed.

For instance, oxidation of alcohol **1o** was found to be low yielding using our conditions and it was hypothesized that micelle formation could be a reason for this sluggish transformation. To test this hypothesis, batch experiments were conducted with and without added sodium dodecyl sulfate (SDS), however, in both cases the desired aldehyde product was formed in ca. 65% after 3 h reaction time at ambient temperature. This data suggest that a longer reaction time is necessary in this case. Flow attempts increasing the residence time (up to 20 min) did not give a significant improvement in the yield. Wirth and co-workers have reported the successful oxidation of geraniol **1p** using (diacetoxy)iodobenzene and a catalytic amount of TEMPO under flow conditions giving the desired aldehyde product with a high conversion (96%) and in an isolated yield of 76%.^22^ In a separate study Hayes and co-workers have used an immobilized TEMPO–NaOCl oxidation and achieved high yields >95% for geraniol.^23^ Unexpectedly, using our TEMPO protocol gave less than 10% of products **2p** and **2q** in a reproducible manner as indicated by ^1^H NMR (ca. 90% remaining substrate). Solubility issues were observed for substrates **1r** and **1s** which prevented their use in flow mode. *N*-Benzylation improved the solubility in case of the modified substrate **1t**, however, no aldehyde formation was observed during the subsequent flow oxidation.

### Robustness Studies

**Scheme 4:**
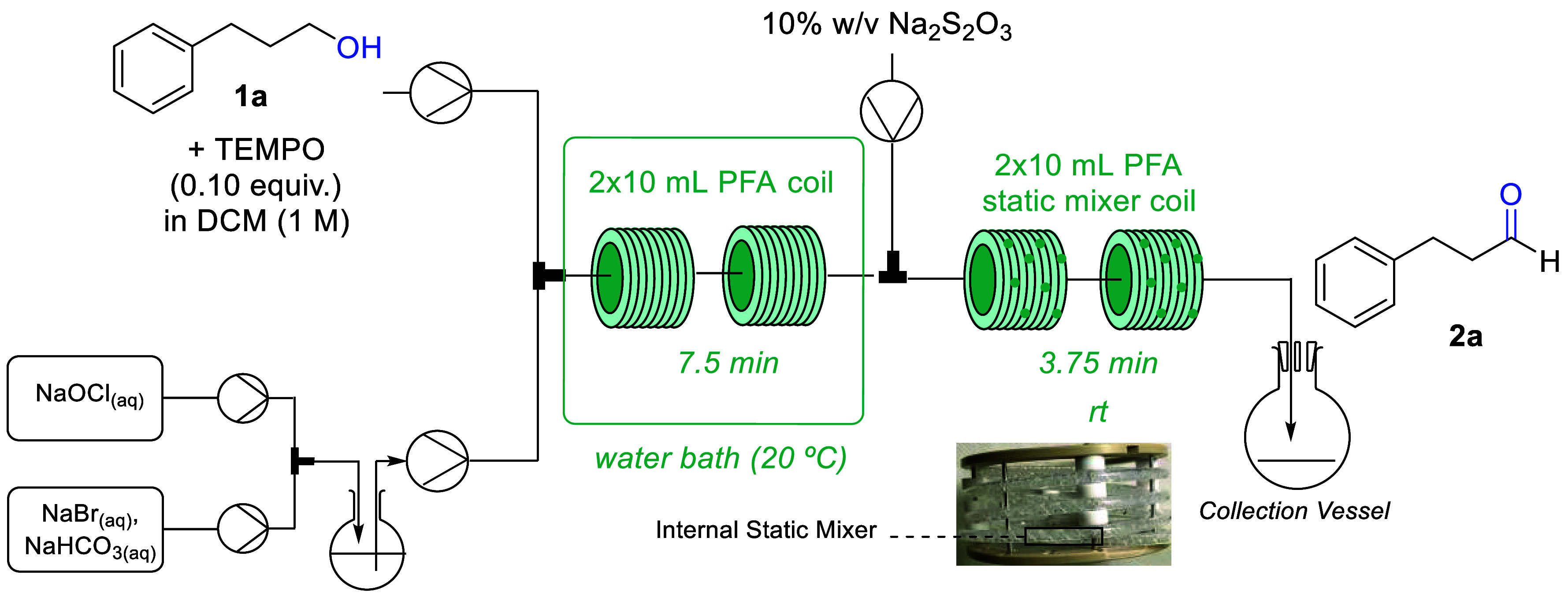
Schematic Set-up of Scale up Process

To evaluate the robustness and scalability of the flow oxidation process a long-run for the conversion of **1a** into phenylpropanal (**2a**) was performed processing 50 g of substrate. To ensure the stability of NaOCl and limit generation of Cl_2_ (via synproportionation of NaOCl), solutions of NaOCl and NaBr/NaHCO_3_ were freshly prepared using a set of syringe pumps as depicted in Scheme 4. The reactor coils were submerged in water baths and the temperature was maintained constant at 20 °C. The reaction mixture was analyzed every 30 min using both GC-FID and ^1^H-qNMR.

**Figure 2:**
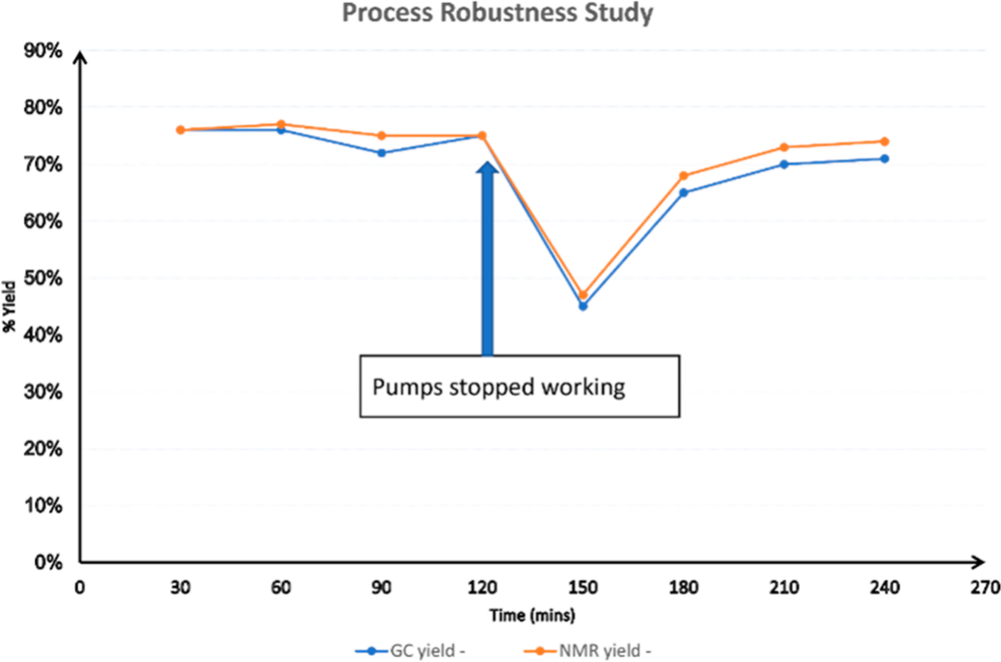
Process robustness over time.

Figure 2 shows the performance of the reaction over time indicating that the yield remained constant (around 75%) for the first 120 min. A distinct drop in performance was noticed at this point which was identified as a problem with one of the pumps. Manual interference quickly resolved this issue and returned high yields for the remainder of this scale-up study. This incidence highlights potential bottlenecks during longer flow processes using regular laboratory equipment and shows that regular sampling is vital to recognize developing faults. Overall, the scale-up of the flow oxidation of phenylpropanol was successful and a throughput of 96 mmol/h was achieved at steady state.

**Scheme 5:**
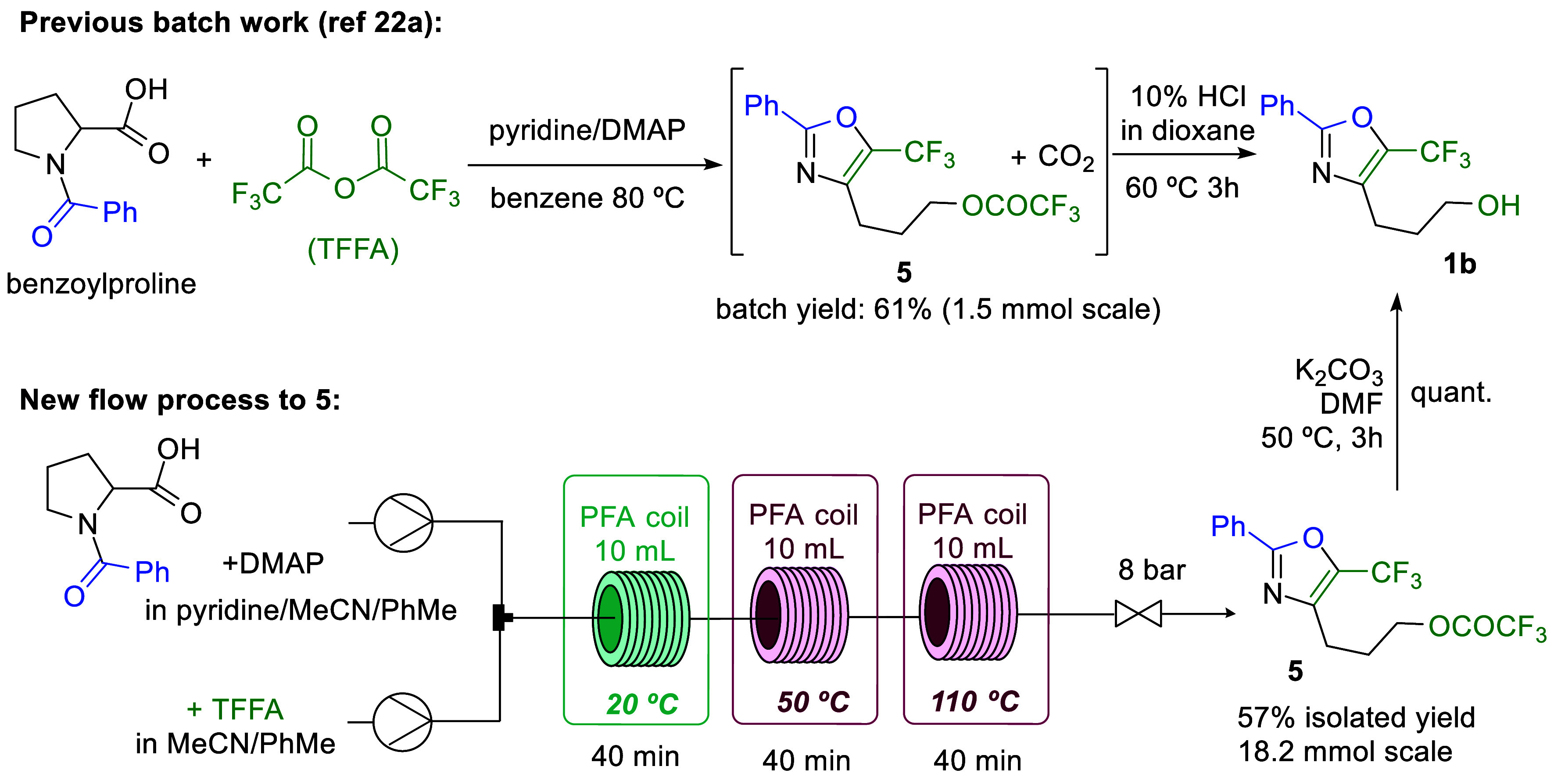
Compound **5** Flow Synthesis and Previous Batch Synthesis

A second example concerned the oxidation of substrate **1b** which contains an unusual trifluoromethyl oxazole. Introduction of an aldehyde functionality on its side chain would provide for further derivatization options to give potentially bioactive molecules. However, as the synthesis of the starting alcohol involved a complex reaction sequence requiring high temperature and different harmful reagents which was difficult to scale in batch, we decided to develop a continuous flow approach for its formation as shown in Scheme 5. The underlying synthesis of substrate **1b** was initially reported by Kawase and co-workers in 1993 and is based on an unconventional reaction between *N*-benzoyl proline and trifluoroacetic anhydride (TFAA) whereby instead of the anticipated Dakin-West product a trifluoromethylated oxazole was obtained by cleavage of the pyrrolidine ring.^24,25^ While some explorations were reported on this process over the years,^25c^ this interesting transformation remains largely forgotten. The original batch reaction requires a long reaction time (8 h), as well as tight control over the rate of addition of TFAA and the temperature and gas release during the reaction. In addition, the reaction conditions in batch require the handling of hazardous materials such as TFAA, a large excess of pyridine and the use of benzene as solvent which made an improved approach for this synthesis desirable. After a short optimization (see SI for more information) a setup with three coiled reactors was used with a progressive increase in temperature to give the best results. Even though the total process time and the yield for the batch and flow approach were found to be similar, both the scalability and reproducibility of this transformation increased providing a productivity of 528 mg/h. In addition, greener solvents, and a safer process for the addition of TFAA resulted from developing the continuous process that ultimately delivered multigram quantities of the desired alcohol after hydrolysis of the trifluoroacetate group of intermediate **5**. Subjecting this material (**1b**) to our flow oxidation process reproduced the initial small-scale results and generated the desired aldehyde **2b** product for further manipulations (76% yield, 14 mmol scale).

**Scheme 6:**
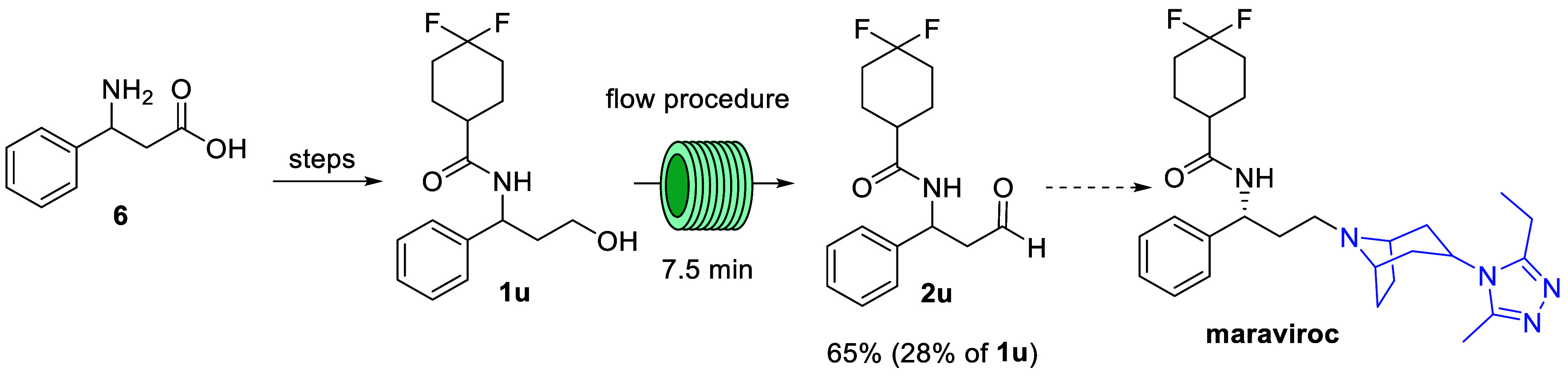
Synthesis of Maraviroc via Key Oxidation Step in Flow

A final application of the developed flow oxidation process targeted the synthesis of aldehyde **2u**, which is a key intermediate in the synthesis of anti-HIV drug maraviroc (Scheme 6).^26^ The alcohol substrate (**1u**) is easily synthesized from 3-amino-3-phenylpropanoic acid according to published procedures. The subsequent oxidation step is described in literature employing Parikh-Doering oxidation conditions. Nevertheless, following this protocol during scale-up generates stoichiometric quantities of dimethyl sulfide which is difficult to contain even after scrubbing. Hence, a revised oxidation of **1u** reports the use of a TEMPO-NaOCl catalyzed oxidation to render a more effective and easier approach. The reported batch protocol at pilot plant scale delivered yields up to 88% for **2u**. However, as this also gave 7–10% of overoxidized product (i.e., carboxylic acid) the procedure requires slow addition of NaOCl with careful control of temperature. When applying our flow-based TEMPO oxidation procedure we decided to limit the conversion of alcohol **1u** to ca. 70% to avoid overoxidation to the corresponding acid which was found to otherwise be a competitively formed product. Under these conditions the desired aldehyde was obtained in an isolated yield of 65%. The unreacted alcohol (ca. 28%) can be reisolated and recycled in subsequent reactions thus minimizing overall loss of material.

## Conclusions

A robust continuous flow process has been developed for the selective oxidation of a wide variety of primary and secondary alcohols. This process uses catalytic amounts of TEMPO as well as NaBr/NaOCl as a simple and cost-effective oxidant system. Throughout this study key parameters such as residence time, reactor type, and temperature were evaluated to obtain effective reaction conditions that rendered a variety of aldehydes and ketones in high chemical yields and within short residence times. An exploratory study also showcased the viability to couple the flow-based oxidation with a continuous extractive separation by converting cyclobutanone into its bisulfite adduct which allows phase separation from remaining starting material and other products. Additionally, process applicability and scalability were trialled by performing multigram scale reaction with the same flow setup. This allowed for the continuous oxidation of 50 g of phenylpropanol, as well as the scale-up of a trifluoromethylated oxazole building block and a precursor toward the HIV drug maraviroc.

## Experimental Section

### General Information

Unless otherwise stated, all solvents were purchased from Fisher Scientific and used without further purification. Substrates and reagents were purchased from Fisher Scientific, Fluorochem, or Sigma-Aldrich and used as-received.

^1^H NMR spectra were recorded on 400 and 500 MHz instruments and are reported relative to residual solvent: CHCl_3_ (δ 7.26 ppm). ^13^C{^1^H}-NMR spectra were recorded on the same instruments (at 100 and 125 MHz) and are reported relative to CHCl_3_ (δ 77.16 ppm). ^19^F-NMR were recorded at 376 MHz. Data for ^1^H NMR are reported as follows: chemical shift (δ/ ppm) (integration, multiplicity, coupling constant (Hz)). Multiplicities are reported as follows: s = singlet, d = doublet, t = triplet, q = quartet, p = pentet, m = multiplet, br. s = broad singlet, app = apparent. Data for ^13^C{^1^H}-NMR are reported in terms of chemical shift (δ/ ppm) and multiplicity (C, CH, CH_2_ or CH_3_). DEPT-135, COSY, HSQC, HMBC, and NOESY experiments were used in the structural assignment. High-resolution mass spectrometry was performed using the indicated techniques on a micromass LCT orthogonal time-of-flight mass spectrometer with leucine-enkephalin (Tyr-Gly-Phe-Leu) as an internal lock mass. GC-MS was performed on a Waters GCT Premier Agilent 7898 system (column Macherey-Nagel; Optima 5 MS, length 15 m, diameter: 0.25 mm). GC-FID: Gas chromatography samples were submitted on Agilent 8860 system (HP-5 column). Continuous flow experiments were performed on a Vaportec E-series system in combination with Chemyx Inc. Fusion 100 syringe pumps for scale-up studies. The flow reactor consisted of 1/16′′ PFA tubing combined with Vaportec reactor coils with internal static mixing elements.

#### General Batch Oxidation Procedure

To a round bottomed flask cooled in a water bath, the alcohol substrate was added and dissolved in DCM to give a 0.25 M – 1 M solution to which 10 mol % TEMPO was added. This was followed by successive addition of 0.6 M NaBr solution (0.23 equiv), NaOCl (1 equiv) and sat. NaHCO_3_ to achieve a pH of ∼9.5. The resulting biphasic reaction mixture was stirred vigorously at ambient temperature and monitored by TLC. After 1 h, 10%w/v Na_2_S_2_O_3_ solution was added to the mixture to quench unreacted NaOCl. The resulting biphasic mixture was washed with DCM and the organic phase was separated and concentrated under reduced pressure providing a crude mixture which was purified by silica gel column chromatography.

#### General Flow Procedure

In a vial (**A**) containing a 1 M solution of the corresponding alcohol in DCM, TEMPO (10 mol %) was dissolved. In a separate vial (**B**) NaOCl (2.21 M, 1 equiv), NaBr (0.6 M, 0.23 equiv) were mixed followed by addition of sat. NaHCO_3_ solution to maintain a pH of 9.5. 10% w/v Na_2_S_2_O_3_ solution was prepared in another vial (**C**) to quench unreacted NaOCl. The reactor coils were flushed with DCM prior to introducing reagents. In a typical flow setup two 10 mL PFA 1/16” reactor coils were connected in series and the outlet is connected to another two 10 mL PFA reactor coils containing an internal static mixer. Solutions from vials (**A**) and (**B**) were pumped at 1.34 mL min^–1^ (combined flow: 2.68 mL min^–1^, residence time: 7.5 min) and mixed within a T-piece mixer. The resulting biphasic stream was then mixed with stream (**C**) pumped at 2.68 mL min^–1^ connected to another 20 mL setup. The resulting organic phase was separated and concentrated under reduced pressure.

#### Long-Run Procedure

In a vial (**A**) containing a 1 M solution of the corresponding alcohol in DCM, TEMPO (10 mol %) was dissolved. A set of syringe pumps were used for dosing to prepare a solution of NaOCl (2.21 M, 1 equiv), NaBr (0.6 M, 0.23 equiv) and sat. NaHCO_3_ which was collected continuously (in vial **B**) to be used for the reaction. 10% w/v Na_2_S_2_O_3_ solution is prepared in another vial (**C**) to quench unreacted NaOCl. The reactor coils were flushed with DCM prior to introducing the reagents. In a typical flow setup two 10 mL PFA 1/16′′ reactor coils were connected in series and the outlet was connected to two further 10 mL PFA reactor coils containing internal static mixers. Solutions from (**A**) and (**B**) were pumped at 1.34 mL min^–1^ (combined flow: 2.68 mL min^–1^, residence time: 7.5 min) and mixed with a T-piece mixer. The resulting biphasic stream is then combined with stream (**C**) pumped at 2.68 mL min^–1^ and directed into another 20 mL setup. The resulting organic phase is separated and concentrated under reduced pressure.

#### Extraction Procedure (Scheme 4)

A general flow procedure was followed for cyclobutanol (**1n**). The resulting outlet stream after reaction quench was collected and the biphasic mixture was separated by gravity separation. The organic layer was pumped at 5 mL min^–1^ and it mixed using a T-piece mixer with another feed containing 10% w/v NaHSO_3_ solution at the same flow rate (combined flow rate: 10 mL min^–1^, residence time: 2 min). The combined biphasic solution was passed through 2 × 10 mL PFA coils with internal static mixer. The biphasic mixture was collected, and phases were separated for further analysis.

Characterization data: Unless otherwise specified, all compounds were obtained following the general conditions of the flow oxidation protocol.

#### 3-Phenylpropanal (2a)

The resulting crude material was purified by flash column chromatography (EtOAc:c-Hex; 1:20) affording the desired compound **2a** as colorless oil in 84% (0.26 g, 2 mmol). ^**1**^**H NMR** (400 MHz, CDCl_3_) δ 9.81 (t, *J* = 1.4 Hz, 1H), 7.30–7.26 (m, 2H), 7.23–7.14 (m, 3H), 2.95 (t, *J* = 7.5 Hz, 2H), 2.77 (tdd, *J* = 7.8, 1.4, 0.7 Hz, 2H). ^**13**^**C NMR** (101 MHz, CDCl_3_) δ 201.5 (CH), 140.3 (C), 128.6 (CH), 128.3 (CH), 126.3 (CH), 45.2 (CH_2_), 28.1 (CH_2_). NMR data is in accordance with a commercial sample.

#### 3-(2-Phenyl-5-(trifluoromethyl)oxazol-4-yl)propanal (2b)

The resulting crude material was purified by flash column chromatography (EtOAc:c-Hex; 1:20) to obtain the desired compound **2b** as colorless solid. Yield: 76% (2.62 g, 9.74 mmol). **Melting range** 57 °C – 59 °C; ^**1**^**H NMR (400 MHz, CDCl**_**3**_**)** δ 9.88 (t, *J* = 1.0 Hz, 1H), 8.31–7.75 (m, 2H), 7.72–7.31 (m, 3H), 3.12–2.99 (m, 2H), 2.97–2.87 (m, 2H); ^**13**^**C NMR (101 MHz, CDCl**_**3**_**)** δ 200.2 (C), 162.1 (C), 141.7 (C, d, *J* = 2 Hz), 134.1 (C, q, *J* = 42 Hz), 131.5 (C), 128.9 (CH), 126.9 (CH), 125.9 (CH), 119.72 (CF_3_, q, *J* = 267 Hz), 41.7 (CH_2_), 18.7 (CH_2_); ^**19**^**F NMR (376 MHz, CDCl**_**3**_**)** δ −61.5 (m); **HRMS** (QTOF) *m*/*z*: [M + H]^+^ Calcd for C_13_H_10_F_3_NO_2_^+^: 270.0736; found 270.0738 (M+H^+^).

#### Dodecanal (2c)

The resulting crude material was purified by flash column chromatography (EtOAc:*n*-pentane; 1:20) to obtain the desired compound **2c** as colorless oil. Yield: 84% (0.16 g, 0.87 mmol). ^**1**^**H NMR (500 MHz, CDCl**_**3**_**)** δ/ppm 9.75 (t, *J* = 1.9 Hz, 1H), 2.40 (td, *J* = 7.4, 1.9 Hz, 2H), 1.62 (p, *J* = 7.3 Hz, 2H), 1.36–1.19 (m, 16H), 0.87 (t, *J* = 7.0 Hz, 3H); ^**13**^**C NMR (126 MHz, CDCl**_**3**_**)** δ/ppm 202.8 (CH), 43.9 (CH_2_), 31.9 (CH_2_), 29.6 (CH_2_), 29.5 (CH_2_), 29.4 (CH_2_), 29.3 (CH_2_), 29.3 (CH_2_), 29.1 (CH_2_), 22.6 (CH_2_), 22.1 (CH_2_), 14.1 (CH_3_); **HRMS** (QTOF) *m*/*z*: [M + H]^+^ Calcd for C_12_H_24_O^+^: 185.1900; found 185.1922 (M+H^+^).

#### Piperonal (2d)

The resulting crude material was purified by flash column chromatography (EtOAc:c-hexane; 1:20) to obtain the desired compound **2d** as colorless solid. Yield: 73% (0.13 g, 0.86 mmol). ^**1**^**H NMR (400 MHz, CDCl**_**3**_**)** δ/ppm 9.80 (d, *J* = 1.1 Hz, 1H), 7.40 (dt, *J* = 7.9, 1.4 Hz, 1H), 7.32 (t, *J* = 1.4 Hz, 1H), 6.91 (dd, *J* = 8.0, 1.1 Hz, 1H), 6.06 (d, *J* = 1.1 Hz, 2H); ^**13**^**C NMR (101 MHz, CDCl**_**3**_**)** δ/ppm 190.3 (CH), 153.1 (C), 148.7 (C), 131.9 (C), 128.6 (CH), 108.3 (CH), 106.9 (CH), 102.1 (CH_2_). NMR data is in accordance with a commercial sample.

#### 2-Iodobenzaldehyde (2e)

The resulting crude material was purified by flash column chromatography (EtOAc:c-hexane; 1:20) to obtain the desired compound **2e** as colorless solid. Yield: 72% (0.12 g, 0.52 mmol). ^**1**^**H NMR (400 MHz, CDCl**_**3**_**)** δ 10.06 (d, *J* = 0.8 Hz, 1H), 7.97–7.91 (m, 1H), 7.87 (dd, *J* = 7.7, 1.8, Hz, 1H), 7.48–7.40 (m, 1H), 7.27 (ddd, *J* = 7.9, 7.3, 1.8 Hz, 1H); ^**13**^**C NMR (126 MHz, CDCl**_**3**_**)** δ/ppm 195.7 (CH), 140.6 (C), 135.4 (CH), 135.1 (CH), 130.2 (CH), 128.7 (CH), 100.6 (C). NMR data is in accordance with a commercial sample.

#### *trans*-Cinnamaldehyde (2f)

The resulting crude material was purified by flash column chromatography (EtOAc:c-hexane; 1:20) to obtain the desired compound **2f** as colorless liquid. Yield: 60% (0.14 g, 1.06 mmol). ^**1**^**H NMR (400 MHz, CDCl**_**3**_**)** δ/ppm 9.68 (dd, *J* = 7.7, 1.1 Hz, 1H), 7.59–7.51 (m, 2H), 7.43–7.38 (m, 4H), 6.69 (ddd, *J* = 16.0, 7.7, 1.1 Hz, 1H); ^**13**^**C NMR (101 MHz, CDCl**_**3**_**)** δ/ppm 193.7 (CH), 152.8 (CH), 134.0 (C), 131.3 (CH), 129.1 (CH), 128.6 (CH), 128.5 (CH). NMR data is in accordance with a commercial sample.

#### 5-(Furan-2-yl)isoxazole-3-carbaldehyde (2g)

The resulting crude material was purified by flash column chromatography (EtOAc:c-hexane; 1:10) to obtain the desired compound **2g** as yellow oil. Yield: 32% (0.11 g, 0.68 mmol). ^**1**^**H NMR (500 MHz, CDCl**_**3**_**)** δ 10.21 (s, 1H), 7.63 (d, *J* = 1.8 Hz, 1H), 7.05 (d, *J* = 3.4 Hz, 1H), 6.83 (s, 1H), 6.62 (dd, *J* = 3.4, 1.6 Hz, 1H); ^**13**^**C NMR (126 MHz, CDCl**_**3**_**)** δ 184.5 (CH), 163.5 (C), 162.3 (C), 145.1 (CH), 142.2 (C), 112.2 (CH), 111.9 (CH), 95.9 (CH); **HRMS** (QTOF) *m*/*z*: [M + H]^+^ Calcd for C_8_H_5_NO_3_^+^: 164.0342; found 164.0344 (M+H^+^).

#### 5-(4-Fluoro-3-(trifluoromethyl)phenyl)isoxazole-3-carbaldehyde (2h)

The resulting crude material was purified by flash column chromatography (EtOAc:c-hexane; 1:5) to obtain the desired compound **2h** as colorless solid. Yield: 71% (0.071 g, 0.27 mmol). **Melting range**: 105 °C – 107 °C; ^**1**^**H NMR (500 MHz, CDCl**_**3**_**)** δ/ppm 10.20 (s, 1H), 8.09 (dd, *J* = 6.5, 2.3 Hz, 1H), 8.06–7.95 (m, 1H), 7.39 (t, *J* = 9.2 Hz, 1H), 6.95 (s, 1H); ^**13**^**C NMR (126 MHz, CDCl**_**3**_**)** δ/ppm 184.3 (CH), 169.4 (C), 162.71 (C), 160.9 (CF, dq, *J* = 262, 2 Hz), 131.6 (CH, d, *J* = 9 Hz), 125.3 (CH, qd, *J* = 5, 2 Hz), 123.1 (C, d, *J* = 4 Hz), 125.2–118.5 (CF, m), 119.8 (C, qd, *J* = 34, 14 Hz), 118.3 (CH, d, *J* = 21 Hz), 97.2 (CH, d, *J* = 1 Hz); ^**19**^**F NMR (470 MHz, CDCl**_**3**_**)** δ/ppm −61.8 (d, *J* = 12 Hz), −109.5–109.6 (m); **HRMS** (QTOF) *m*/*z*: [M + H]^+^ Calcd for C_11_H_5_F_4_NO_2_^+^: 260.0329; found 260.0329 (M+H^+^).

#### 5-(2,4-Dichloro-5-fluorophenyl)isoxazole-3-carbaldehyde (2i)

In this case, the general procedure was followed using a different concentration: 0.25 M for the alcohol and 0.32 M for NaOCl. The resulting crude material was purified by flash column chromatography (EtOAc:c-hexane; 1:5) to obtain the desired compound **2i** as colorless solid. Yield: 57% (0.075 g, 0.29 mmol). **Melting range**: 137 °C – 139 °C; ^**1**^**H NMR (500 MHz, CDCl**_**3**_**)** δ/ppm 10.21 (s, 1H), 7.81 (d, *J* = 9.3 Hz, 1H), 7.62 (d, *J* = 6.6 Hz, 1H), 7.37 (s, 1H); ^**13**^**C NMR (126 MHz, CDCl**_**3**_**)** δ/ppm 184.3 (CH), 166.3 (C, d, *J* = 2 Hz), 162.6 (C), 156.9 (CF, d, *J* = 251 Hz), 132.7 (CH), 127.3 (C, d, *J* = 4 Hz), 124.9 (C, d, *J* = 7 Hz), 124.5 (C, d, *J* = 19 Hz), 116.8 (CH, d, *J* = 25 Hz), 101.9 (CH); ^**19**^**F NMR (470 MHz, CDCl**_**3**_**)** δ/ppm −115.1 (dd, *J* = 9, 6 Hz); **HRMS** (QTOF) *m*/*z*: [M + H]^+^ Calcd for C_10_H_4_Cl_2_FNO_2_^+^: 259.9676; found 259.9676 (M+H^+^).

#### Benzophenone (2j)

The resulting crude material was purified by flash column chromatography (EtOAc:c-hexane; 1:20) to obtain the desired compound **2j** as a colorless solid. Yield: 75% (0.12 g, 0.65 mmol). ^**1**^**H NMR (400 MHz, CDCl**_**3**_**)** δ/ppm 7.82–7.76 (m, 4H), 7.62–7.54 (m, 2H), 7.51–7.43 (m, 4H); ^**13**^**C NMR (101 MHz, CDCl**_**3**_**)** δ/ppm 196.7 (C), 137.6 (C), 132.4 (CH), 130.0 (CH), 128.3 (CH). NMR data is in accordance with a commercial sample.

#### 1-(2,4-Difluorophenyl)-3-(pyridin-2-yl)propan-1-one (2k)

The resulting crude material was purified by flash column chromatography (EtOAc:c-hexane; 1:4) to obtain the desired compound **2k** as colorless liquid. Yield: 58% (0.097 g, 0.39 mmol). ^**1**^**H NMR (400 MHz, CDCl**_**3**_**)** δ/ppm 8.48–8.46 (m, 1H), 7.90 (td, *J* = 8.6, 6.6 Hz, 1H), 7.56 (td, *J* = 7.6, 1.9 Hz, 1H), 7.23–7.20 (m, 1H), 7.09–7.06 (m, 1H), 6.97–6.88 (m, 1H), 6.83 (ddd, *J* = 11.1, 8.7, 2.4 Hz, 1H), 3.45 (td, *J* = 7.1, 3.3 Hz, 2H), 3.20 (t, *J* = 7.1 Hz, 2H)f; ^**13**^**C NMR (101 MHz, CDCl**_**3**_**)** δ/ppm 196.1 (C, d, *J* = 4 Hz), 165.6 (CF, dd, *J* = 293, 12 Hz), 163.0 (CF, *J* = 294, 12 Hz), 160.6 (C), 149.3 (CH), 136.4 (CH), 132.7 (CH, dd, *J* = 10, 4 Hz), 123.3 (CH), 122.3 (C, dd, *J* = 13, 3 Hz), 121.3 (CH), 112.2 (CH, dd, *J* = 21, 3 Hz), 104.8 (CH, dd, *J* = 28, 25 Hz), 42.5 (CH_2_, d, *J* = 8 Hz), 32.0 (CH_2_, d, *J* = 2 Hz); ^**19**^**F NMR (376 MHz, CDCl**_**3**_**)** δ/ppm −102.0 (m), −103.9 (m); **HRMS** (QTOF) *m*/*z*: [M + H]^+^ Calcd for C_14_H_11_F_2_NO^+^: 248.0881; found 248.0905 (M+H^+^).

#### 1-(Thiazol-2-yl)ethan-1-one (2l)

The resulting crude material was purified by flash column chromatography (EtOAc:c-hexane; 1:15) to obtain the desired compound **2l** as colorless liquid. Yield: 80% (0.089 g, 0.70 mmol). ^**1**^**H NMR (500 MHz, CDCl**_**3**_**)** δ/ppm 8.01 (t, *J* = 2.9 Hz, 1H), 7.68 (dd, *J* = 3.0, 1.8 Hz, 1H), 2.73 (d, *J* = 3.1 Hz, 3H); ^**13**^**C NMR (126 MHz, CDCl**_**3**_**)** δ/ppm 191.7 (C), 167.3 (C), 144.8 (CH), 126.4 (CH), 26.0 (CH_3_); **HRMS** (QTOF) *m*/*z*: [M + H]^+^ Calcd for C_5_H_5_SNO^+^: 128.0165; found 128.0167 (M+H^+^).

#### Cyclopentanone (2m)

The resulting crude material was purified by flash column chromatography (EtOAc:*n*-pentane; 1:20) to obtain the desired compound **2m** as colorless oil. Yield: 69% (0.26 g, 3.22 mmol). ^**1**^**H NMR (400 MHz, CDCl**_**3**_**)** δ/ppm 2.23–2.01 (m, 4H), 1.91–1.87 (m, 4H); ^**13**^**C NMR (101 MHz, CDCl**_**3**_**)** δ/ppm 220.5 (C), 38.3 (CH_2_), 23.1 (CH_2_). NMR data is in accordance with a commercial sample.

#### 3,7-Dimethyloctanal (2o)

The resulting crude material was purified by flash column chromatography (EtOAc:*n*-pentane, 1:40) to obtain the desired compound **2o** as colorless oil. Yield: 10% (0.02 g). ^**1**^**H NMR (500 MHz, CDCl**_**3**_**)** δ/ppm 9.75 (t, *J* = 2.4 Hz, 1H), 2.39 (ddd, *J* = 16.0, 5.7, 2.1 Hz, 1H), 2.22 (ddd, *J* = 16.0, 7.9, 2.6 Hz, 1H), 2.11–1.98 (m, 1H), 1.52 (dp, *J* = 13.2, 6.7 Hz, 1H), 1.35–1.11 (m, 6H), 0.96 (d, *J* = 6.7 Hz, 3H), 0.87–0.85 (m, 6H); ^**13**^**C NMR (126 MHz, CDCl**_**3**_**)** δ/ppm 203.2 (CH), 51.1 (CH_2_), 39.0 (CH_2_), 37.1 (CH_2_), 28.2 (CH), 27.9 (CH), 24.7 (CH_2_), 22.6 (CH_3_), 22.5 (CH_3_), 20.0 (CH_3_). Data is in accordance with the literature.^27^

#### 4,4-Difluoro-*N*-(3-oxo-1-phenylpropyl)cyclohexane-1-carboxamide (2u)

The resulting crude material was purified by flash chromatography (EtOAc:c-hexane, 1:5) to obtain the desired compound **2u** as colorless solid. Yield: 65% (0.1 g, 0.34 mmol). **Melting range**: 118 °C – 120 °C; ^**1**^**H NMR (500 MHz, CDCl**_**3**_**)** δ/ ppm 9.75 (dd, *J* = 2.5, 1.1 Hz, 1H), 7.37–7.32 (m, 2H), 7.31–7.26 (m, 3H), 6.26 (br d, *J* = 8.2 Hz, 1H), 5.67–5.31 (m, 1H), 3.05 (ddd, *J* = 16.7, 7.0, 2.5 Hz, 1H), 2.96 (ddd, *J* = 16.8, 5.7, 1.2 Hz, 1H), 2.21–2.09 (m, 3H), 1.95–1.87 (m, 2H), 1.86–1.64 (m, 4H). ^**13**^**C NMR (126 MHz, CDCl**_**3**_**)** δ/ ppm 200.3 (CH), 173.5 (C, d, *J* = 2 Hz), 140.2 (C), 129.0 (CH), 128.0 (CH), 126.4 (CH), 125.0–120.0 (CF_2_, m), 48.9 (CH), 48.4 (CH_2_), 42.7 (CH_2_), 33.6–32.3 (CH_2_, m), 25.8 (CH_2_, ddd, *J* = 12, 9, 1 Hz). ^**19**^**F NMR (470 MHz, CDCl**_**3**_**)** δ/ ppm −93.2 (d, *J* = 237 Hz), −100.9 (d, *J* = 236 Hz). **HRMS** (QTOF) *m*/*z*: [M + H]^+^ Calcd for C_16_H_19_F_2_NO_2_^+^: 296.1457; found 296.1457 (M+H^+^). Data is in accordance with the literature.^23^

#### 3-(2-Phenyl-5-(trifluoromethyl)oxazol-4-yl)propyl 2,2,2-trifluoroacetate (5)

##### Long-Run Procedure in Flow

In a vial (**A**), *N*-benzoyl proline (4 g, 18.24 mmol, 1 equiv) DMAP (446 mg, 3.6 mmol, 0.12 equiv) and pyridine (13.5 mL, 164.16 mmol, 9 equiv) were dissolved in toluene:MeCN (2:1) to give a volume of 45.6 mL (0.4 M). In a separate vial (**B**) trifluoroacetic anhydride (15.22 mL, 109.46 mmol, 6 equiv) were dissolved in toluene:MeCN (2:1, final volume 45.6 mL). The reactor coils were flushed with the mixture of acetonitrile/toluene prior to introducing reagents. After conditioning the three coils at their corresponding temperature (r.t., 50 °C, 110 °C, 7 bar) for 10 min, both solutions were filled in the injection ports. Solutions from (**A**) and (**B**) were pumped at 125 μL min^–1^ (combined flow: 250 μL min^–1^, residence time: 120 min, total time: 7 h) and mixed within a T-piece mixer at r.t. Once all the solutions were pumped into the system, a solution of toluene/acetonitrile was placed in the inlet of both feeds. The reaction mixture was collected in a flask and concentrated in vacuo. The resulting crude material was purified by flash column chromatography (EtOAc:c-Hex; 1:10) to obtain the desired compound **5** (3.7 g, 57% yield) as colorless oil. ^**1**^**H NMR** (400 MHz, CDCl_3_) δ 8.05 (m, 2H), 7.54–7.43 (m, 3H), 4.43 (t, *J* = 6.3 Hz, 2H), 2.89–2.81 (m, 2H), 2.29–2.11 (m, 2H); ^13^**C NMR** (101 MHz, CDCl_3_) δ 162.3 (C), 157.4 (C, q, *J* = 42 Hz), 141.8 (C, q, *J* = 2 Hz), 134.5 (C, q, *J* = 42 Hz), 131.6 (C), 129.0 (CH), 127.0 (CH), 125.9 (CH), 119.7 (CF_3_, q, *J* = 267 Hz), 111.6 (CF_3_, q, *J* = 286 Hz), 66.8 (CH_2_), 26.7 (CH_2_), 22.0 (CH_2_); ^**19**^**F NMR** (376 MHz, CDCl_3_) δ −61.6, −75.2; **HRMS** (QTOF) *m*/*z*: [M + H]^+^ Calcd for C_15_H_11_F_6_NO_3_^+^: 368.0716; found 368.0718 (M+H^+^).

#### 3-(2-Phenyl-5-(trifluoromethyl)oxazol-4-yl)propan-1-ol (1b)

In a 25 mL round bottomed flask a solution of **5** in DMF (0.27 M) was prepared followed by addition of K_2_CO_3_ (0.3 g, 2.17 mmol, 4 equiv). The resulting reaction mixture was stirred for 3 h at 50 °C. The reaction mixture was then quenched with ice cold water (5 mL) followed by extraction with EtOAc (3 × 20 mL). The organic layer was separated and evaporated under reduced pressure giving **1b** in quantitative yields (0.14 g) as a colorless solid. **Melting range**: 58 °C – 60 °C; ^**1**^**H NMR**: **(400 MHz, CDCl**_**3**_**)** δ 8.09–7.98 (m, 2H), 7.55–7.40 (m, 3H), 3.74 (t, *J* = 6.1 Hz, 2H), 2.98–2.76 (m, 2H), 2.46 (s, 1H), 2.11–1.87 (m, 2H); ^**13**^**C NMR**: **(101 MHz, CDCl**_**3**_**)** δ 162.0 (C), 143.1 (C, q, *J* = 2 Hz), 134.2 (C, d, *J* = 42 Hz), 131.6 (C), 128.9 (CH), 126.9 (CH), 125.9 (CH), 119.8 (CF_3_, q, *J* = 267 Hz), 61.7 (CH_2_), 31.1 (CH_2_), 22.6 (CH_2_); ^**19**^**F NMR: (376 MHz, CDCl**_**3**_**)** δ −61.5; **HRMS** (QTOF) *m*/*z*: [M + H]^+^ Calcd for C_13_H_12_F_3_NO_2_^+^: 272.0893; found 272.0892 (M+H^+^).

## Supporting Information Available

Additional optimization tables for continuous synthesis of 5, additional flow oxidation graphs and tables; pictures of the flow equipment setup, and copies of the NMR spectra (PDF)
