# Supplementary material for: Continuous Flow Oxidation of Alcohols Using TEMPO/NaOCl for the Selective and Scalable Synthesis of Aldehydes
Source: Org Process Res Dev. 2023 Sep 7;28(5):1587–96. doi: 10.1021/acs.oprd.3c00237 (PMC11110051; doi:10.1021/acs.oprd.3c00237)
Supplement: Supplementary file 1 — op3c00237_si_001.pdf [file op3c00237_si_001.pdf]

Supporting Information

Continuous Flow Oxidation of Alcohols Using TEMPO/NaOCl for the Selective and Scalable Synthesis of Aldehydes

Parth Naik,[a] Jorge Garcia-Lacuna,[a] Patrick O'Neill,[b] and Marcus Baumann[a,*]

[a] School of Chemistry, University College Dublin, Science Centre South, Belfield, D04 N2E5, Ireland.
[b] Pfizer Ireland, Ringaskiddy; P43 X336; Ireland
*Email: marcus.baumann@ucd.ie

Table of Contents:

1. **Optimization Studies of the continuous flow oxidation**…………………………………...S3
2. **Flow synthesis optimization for oxazole 5** ..……………………………………………….S6
3. **Pictures of the flow equipment**…………………………………………………..…….S9
4. **Copies of NMR spectra**
    a. **NMR spectra of oxidation products (2a-2m, 2o and 2u)**…………………………S10
    b. **NMR spectra of oxazole products (1b and 5)**………………...……………….S30

1. Optimization studies of the continuous flow oxidation

- Yield vs Temperature comparison using different heating/cooling methods.

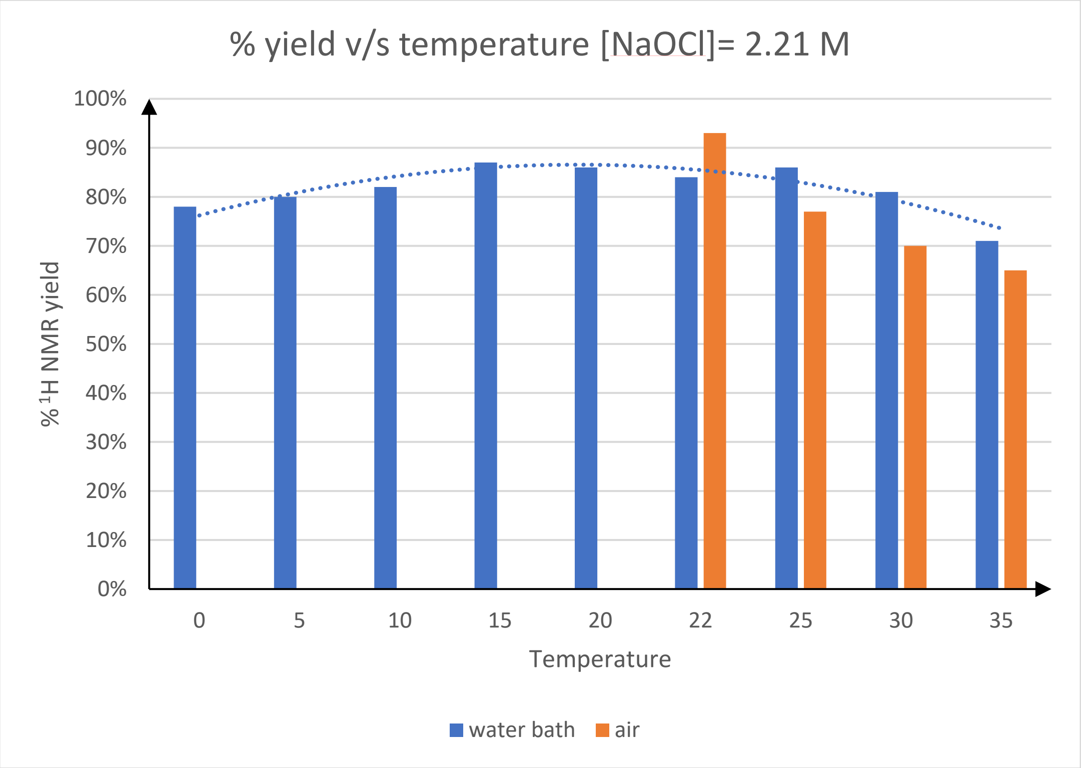

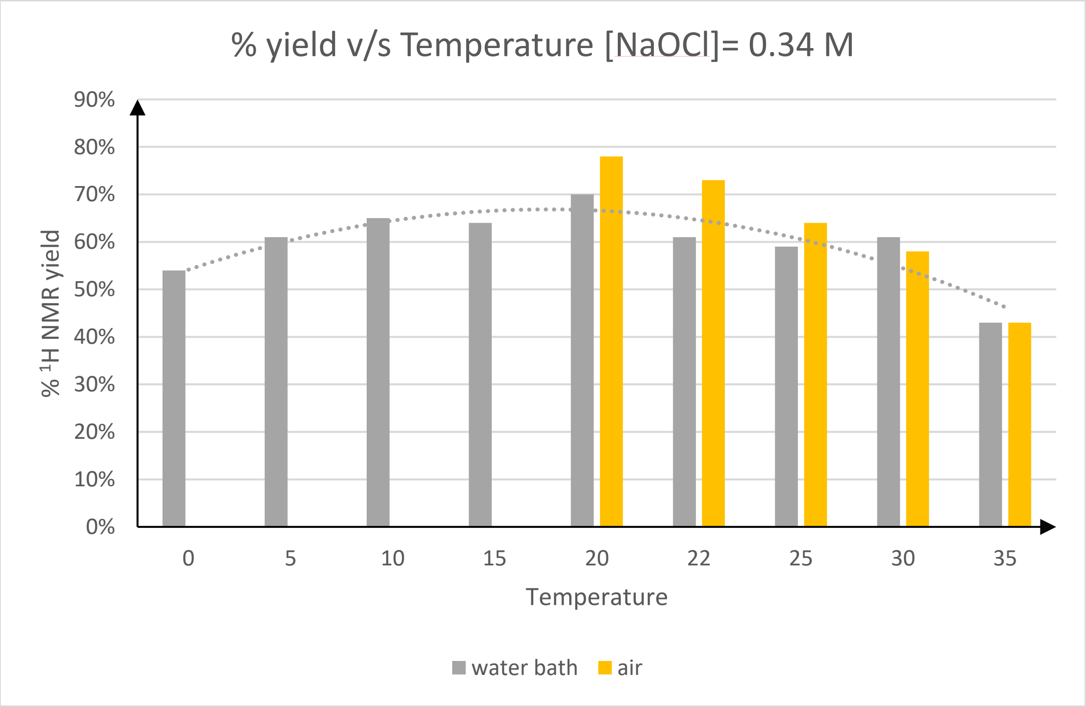

Figure S1: % yield v/s temperature for different quality of NaOCl.

- Study of different pumps for the continuous flow oxidation of **1a**

Table S*1*: %yield for different types of pumps

**Type of pump**	**Reactor vol. (ml)**	**Total flow rate (ml/min)**	**% Yield (by ^{1}H NMR)[a]**
Syringe pumps	10	1.33	80%
Piston pumps	10	1.33	85%
Peristaltic pumps	10	1.33	86%

General conditions: Using two sets of pumps T = 17 °C, Feed 1: conc. [NaOCl] = 0.34 M, and NaBr (0.23 equiv.) TEMPO equiv: 0.10, Feed 2: alcohol 1a in DCM (0.25 M) a) Calculated by qNMR using 1,3,5-trimethoxybenzene as an internal standard.

Cyclobutanol (**1n**) oxidation optimization.

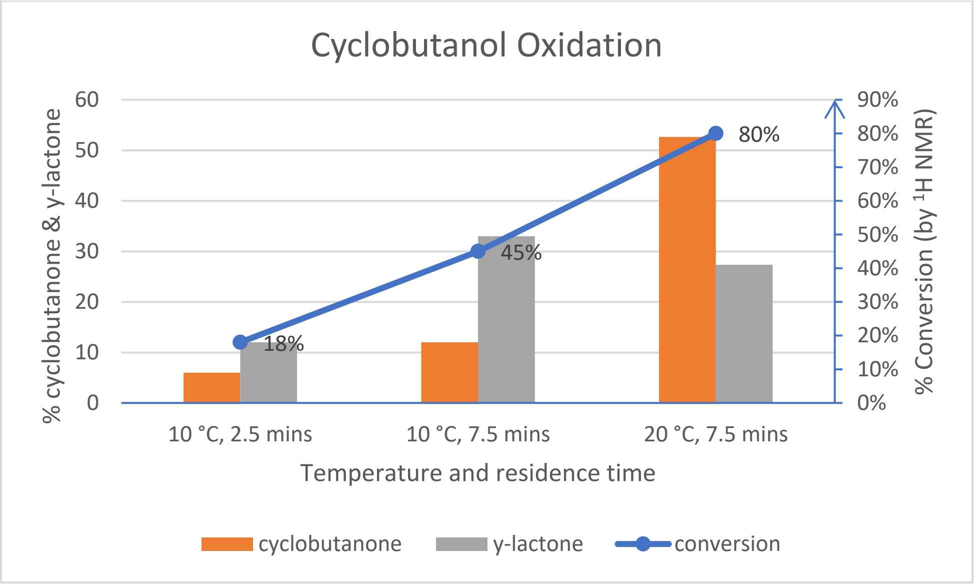

Figure S2: Conversion of cyclobutanol v/s temperature v/s residence time.

- NMRs comparison after extractive work-up

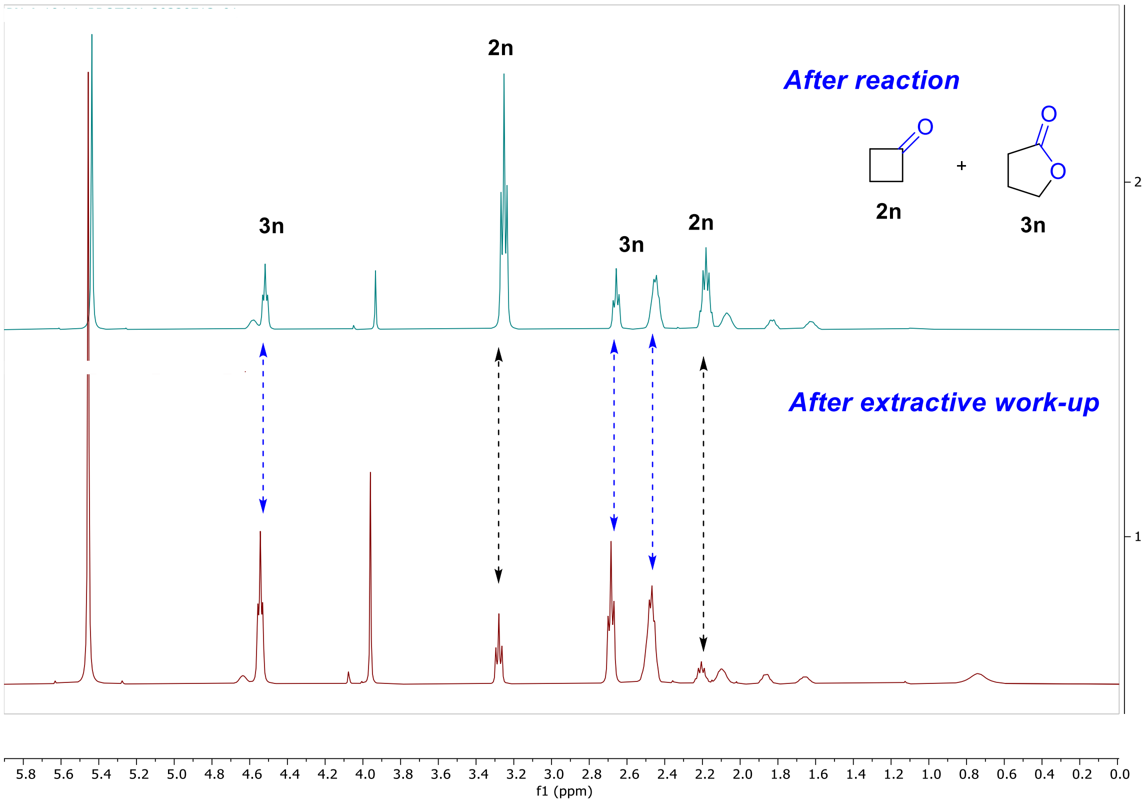

Figure S3: ^{1}H NMR spectrum comparison for cyclobutanone (2o) & lactone (3o).

2. Flow synthesis optimization for oxazole 5

- Initial Set-up

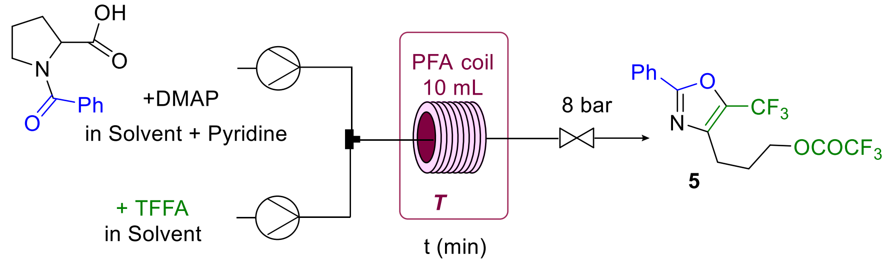

Table S2: Flow optimization of **5** syntheses using 1 coil set-up.

Entry[a]	Res. Time (min)	Pyridine equiv.	TFFA equiv.	Temperature (ºC)	NMR yield[b]
1	10	6	4	90	5%
2	20	6	4	80	-
3	20	6	4	90	10%
4	20	6	4	100	13%
5	20	6	4	110	12%
6[c]	20	6	4	100	6%
7	20	9	4	100	13%
8	20	9	6	100	20%
9	60	9	6	100	27%
10[d]	60	9	6	100	15%
11[e]	30+30	9	6	20+100	47%

a) All reactions were performed at 0.4 mmol scale with two feeds. Feed 1: N-benzoyl proline, *DMAP, (0.12 equiv.) and pyridine (x equiv.) in Toluene:MeCN (2:1; 0.4M). Feed 2: Trifluoroacetic anhydride (y equiv.) in Toluene:MeCN (2:1); b) 1,3,5 Trimethoxybenzene was used as internal standard; c) Reaction with the T-piece and the tube before the reactor submerged in an ice bath d) Reaction in absence of DMAP; e) Reaction using two coils at different temperatures*

- Three coil set-up

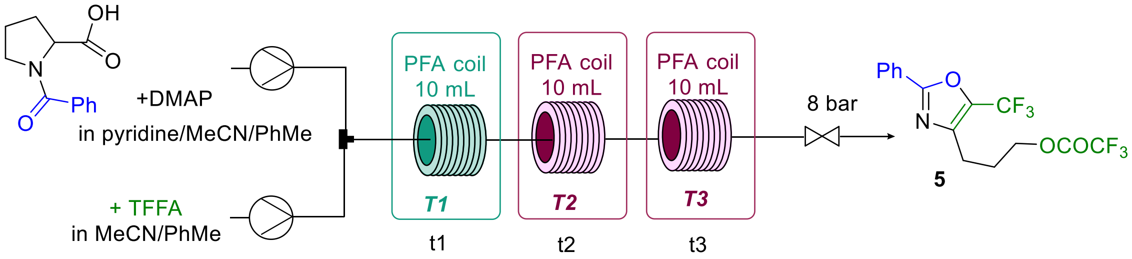

Table S3: Flow optimization of **5** syntheses using 3 coils set-up.

Entry[a]	Time in each reactor (min)	T 1 (°C)	T 2 (°C)	T 3 (°C)	NMR yield[b]	Isolated yield
1	30	20	100	100	38%	
2	30	20	30	100	48%	34%
3	30	20	50	100	57%	46%
4	15	20	50	100	43%	
5	40	20	30	110	58%	51%
6	40	20	50	110	68%	61%
7	40	20	50	120	58%	46%
8[c]	40	20	50	110	64%	57%

a) All reactions were performed at 0.4 mmol scale with two feeds. Feed 1: N-benzoyl proline, DMAP, (0.12 equiv.) and *pyridine (9 equiv.) in toluene:MeCN (2:1; 0.4M). Feed 2: Trifluoroacetic anhydride (6 equiv.) in toluene:MeCN (2:1). b) 1,3,5-trimethoxybenzene was used as internal standard; c) Scale-up experiment at 18.2 mmol scale. Best conditions (used for the long-run are highlighted in color)*

Note that to get better isolated yields the chromatography was performed immediately after the reaction; product decomposition (5-10%) was found as an inevitable side-effect during chromatography.

3. Pictures of the flow equipment and chosen set-ups

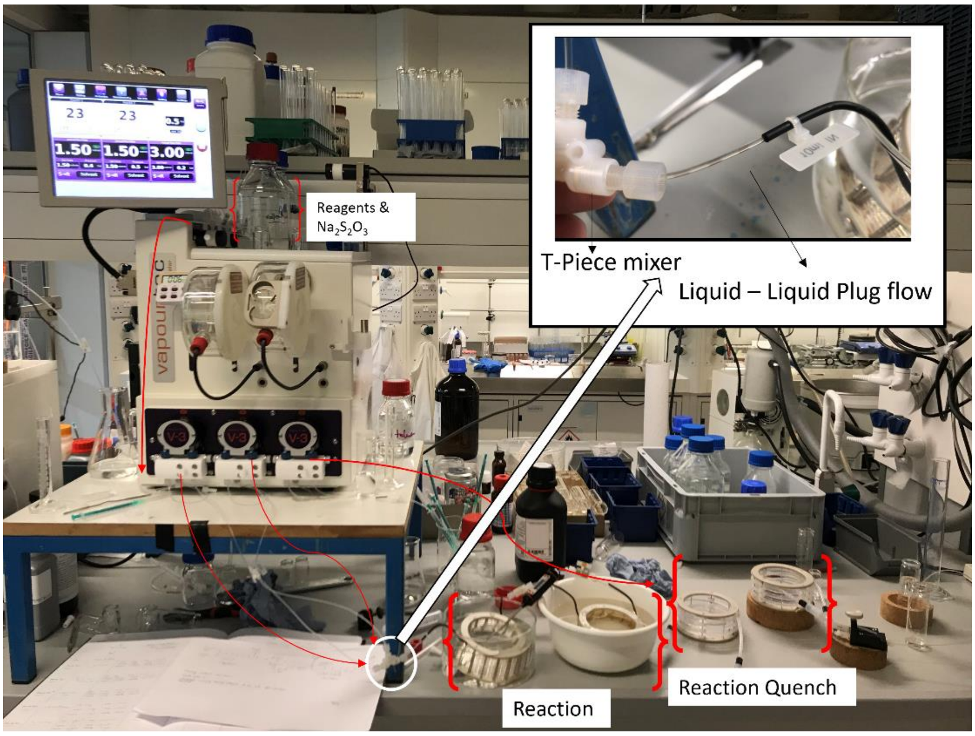

Figure S4: *Telescoped continuous extractive workup setup in lab*

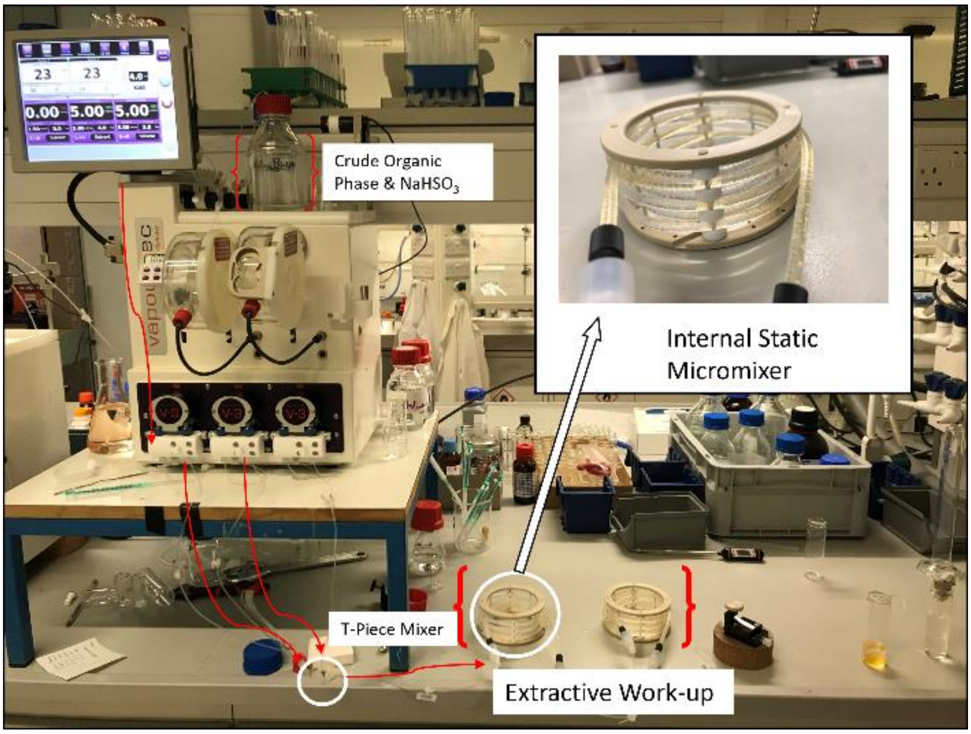

Figure S5: *Liquid-Liquid plug flow*

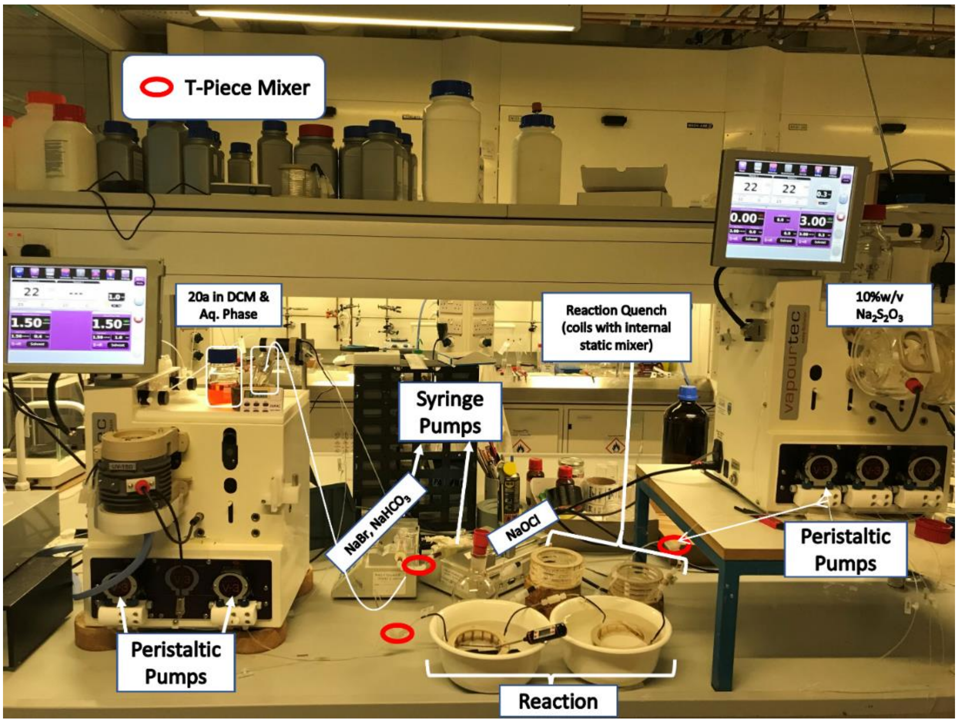

Figure S6: *Setup for multigram scaleup flow process.*

4. Copies of NMR Spectra.

3-Phenylpropanal **(2a):**

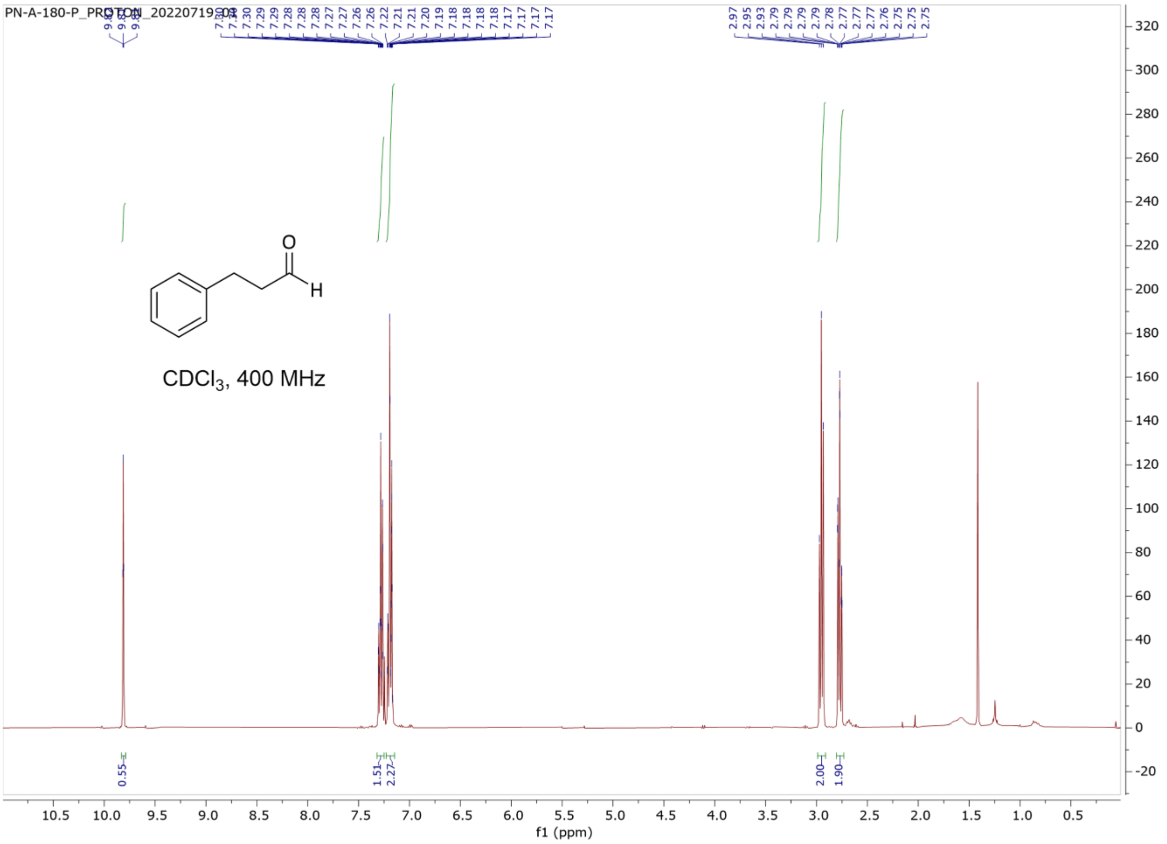

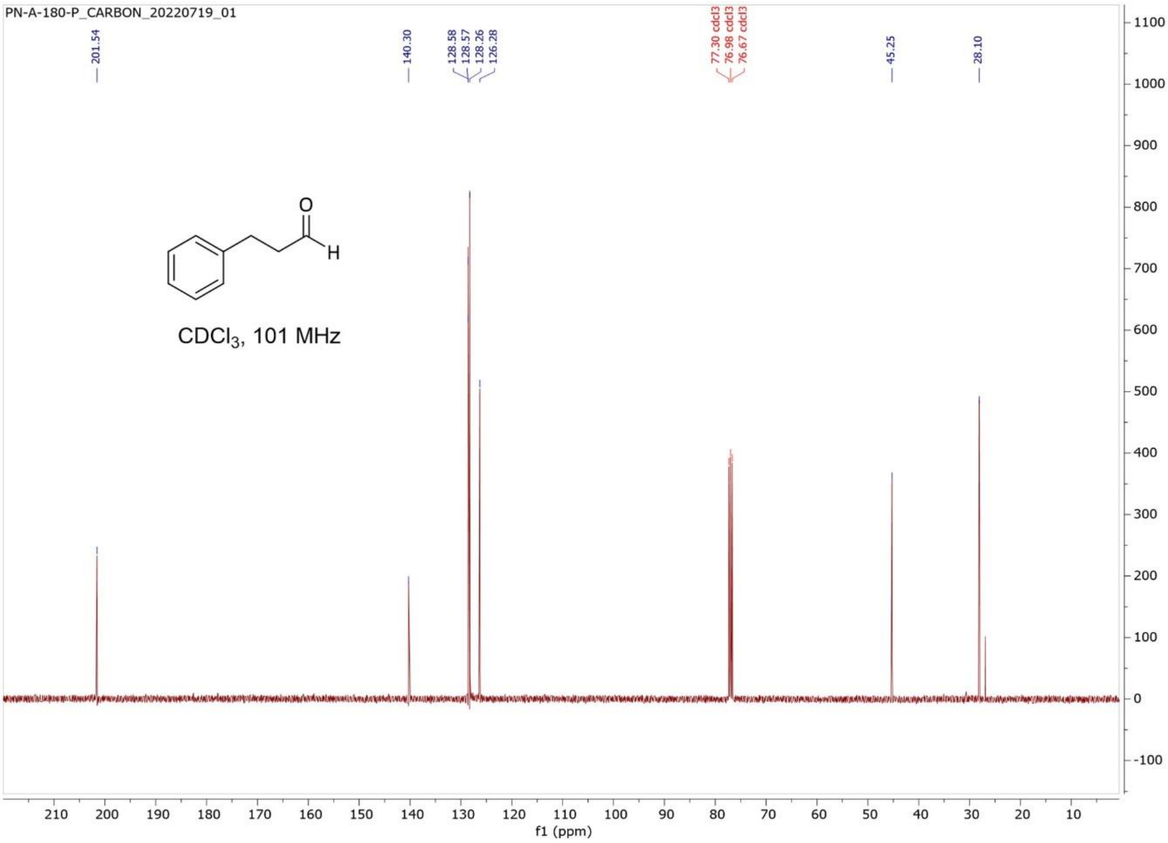

3-(2-Phenyl-5-(trifluoromethyl)oxazol-4-yl)propanal **(2b):**

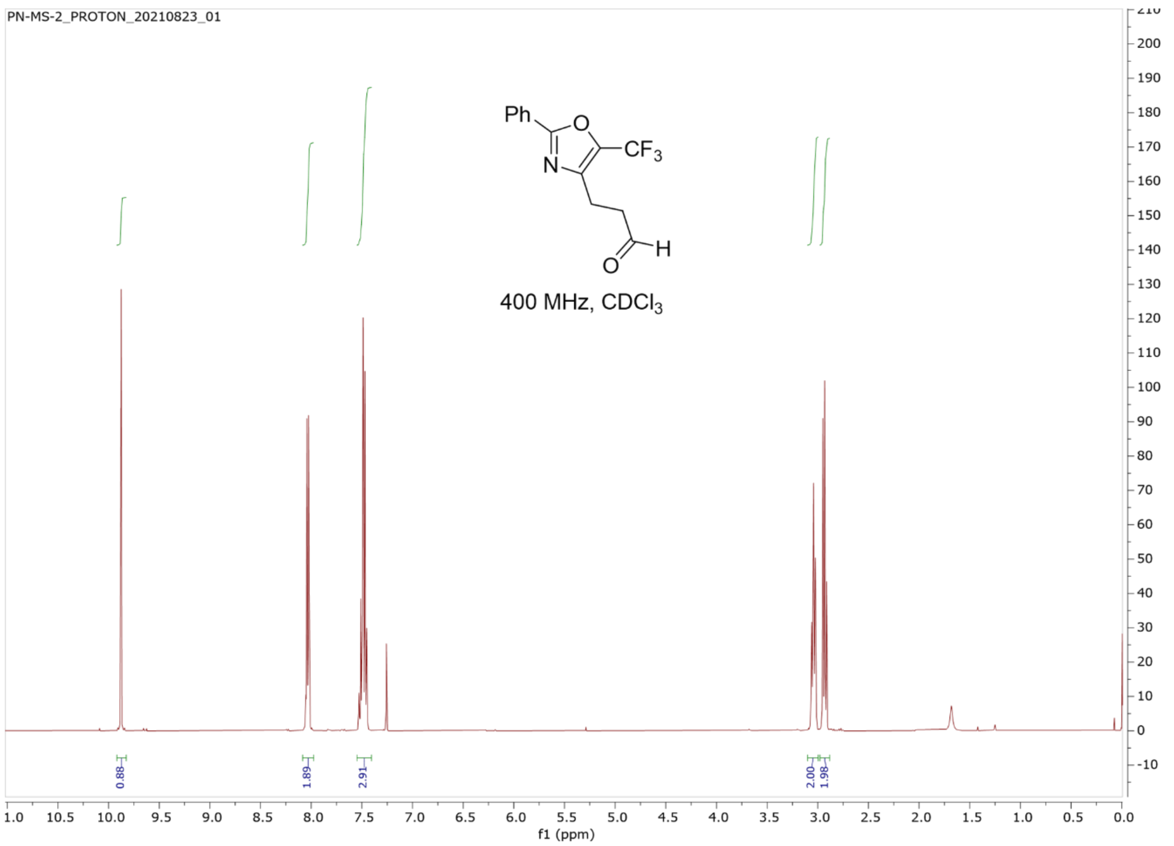

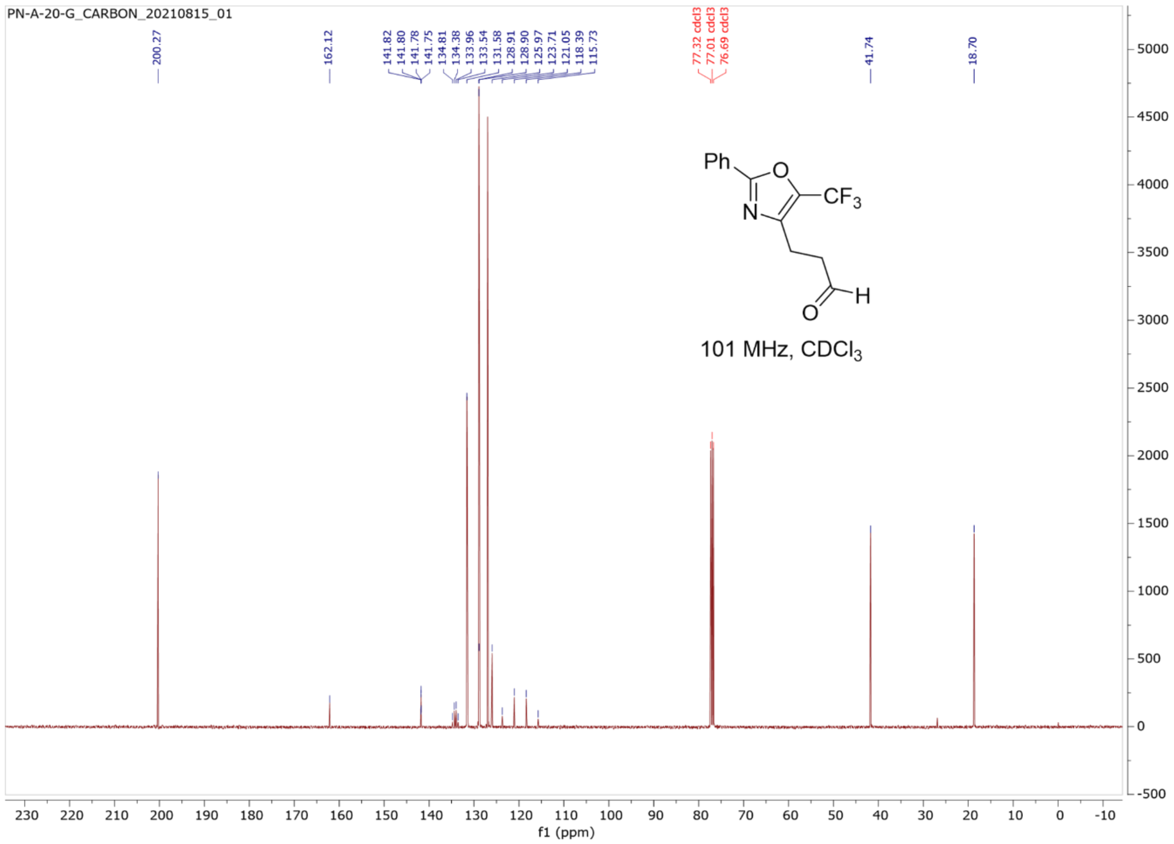

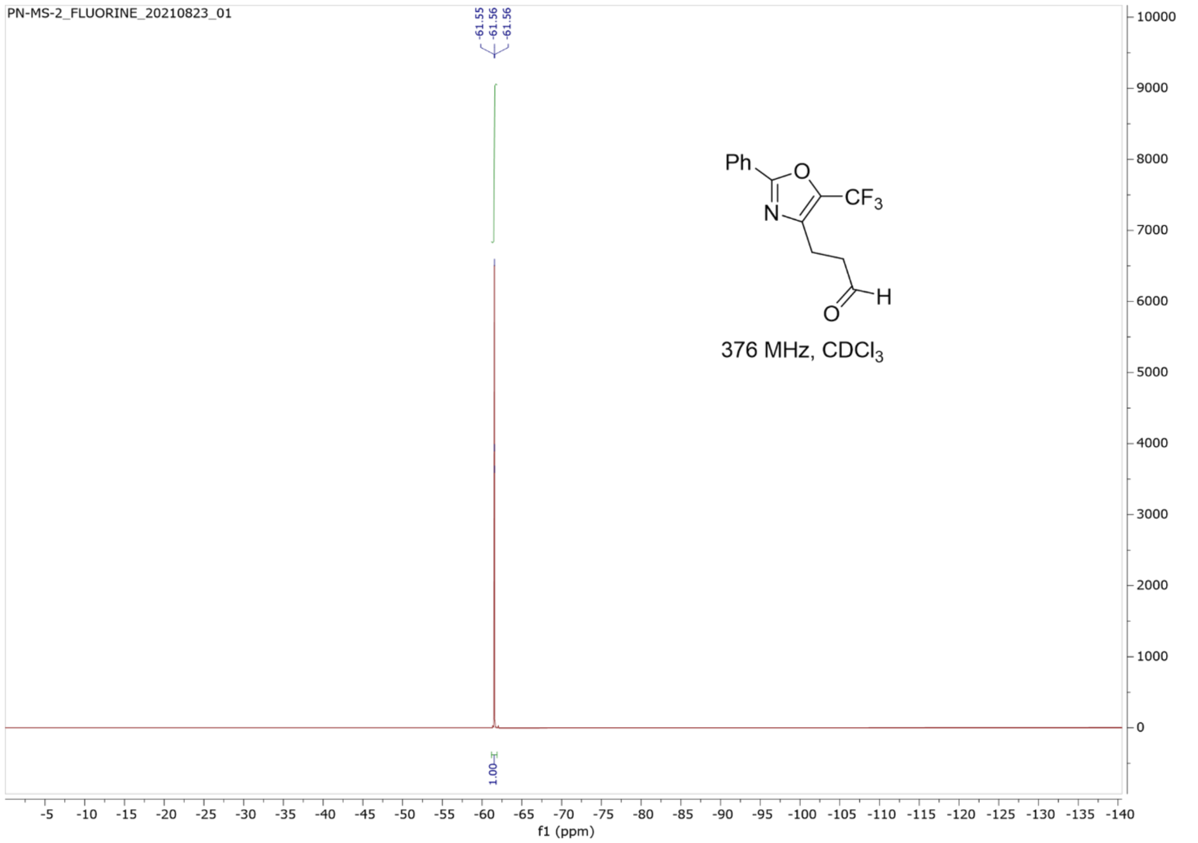
PN-MS-2_FLUORINE_20210823_01
-61.55
-61.56
-61.56
Ph
O
N
CF3
H
O
376 MHz, CDCl3
1.00
f1 (ppm)

Dodecanal **(2c):**

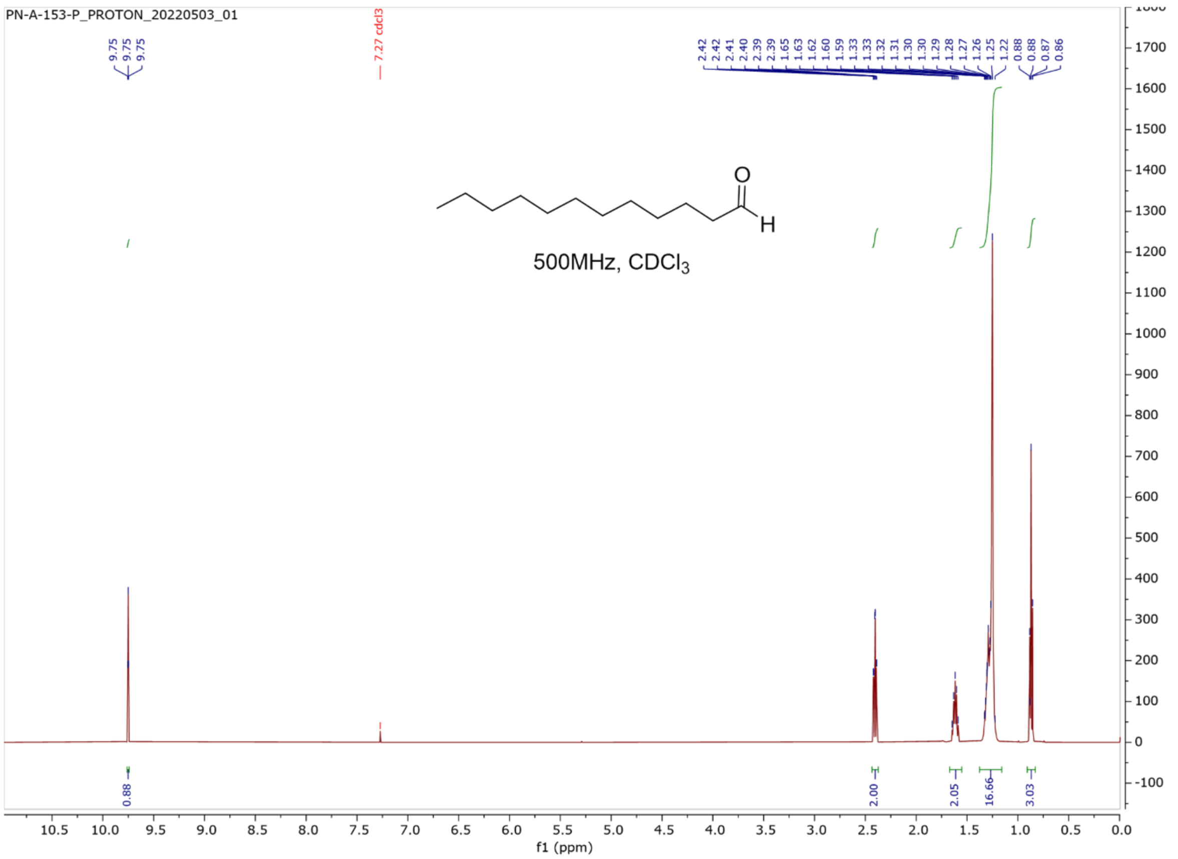

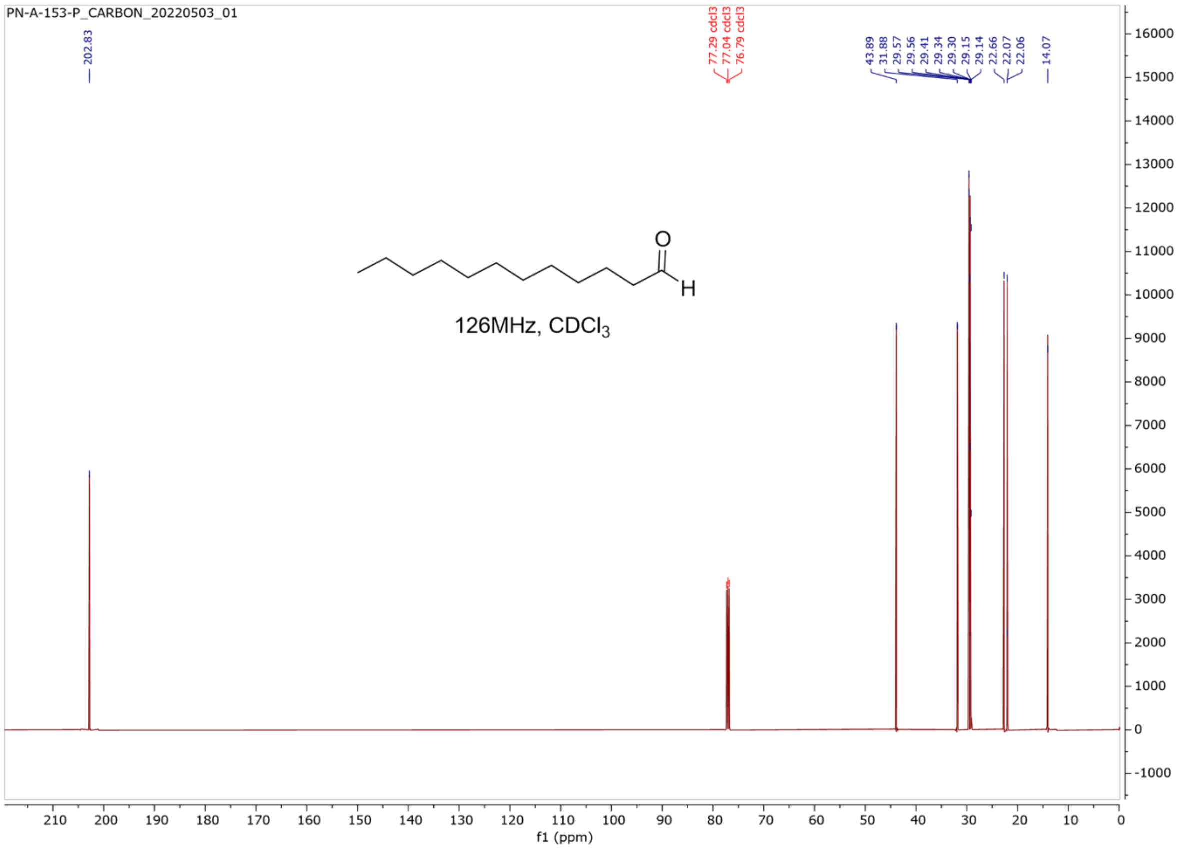

Piperonal **(2d):**

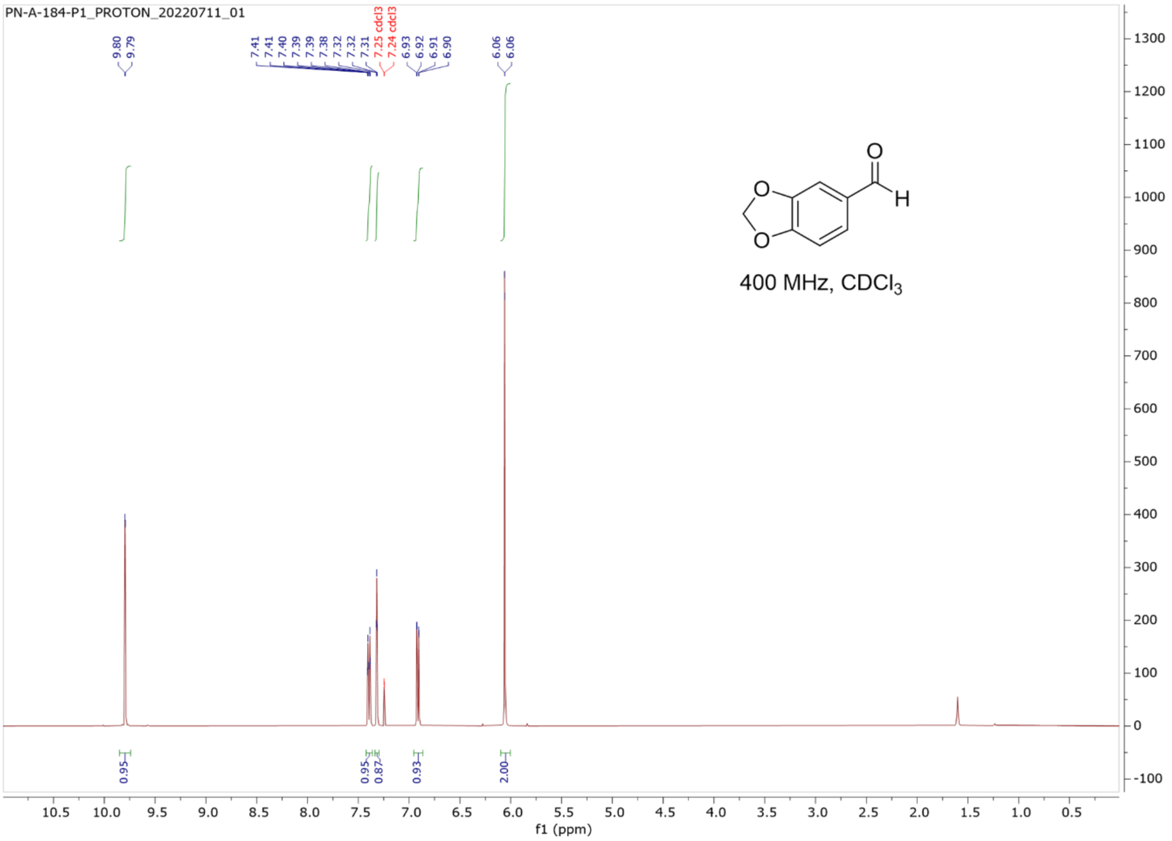

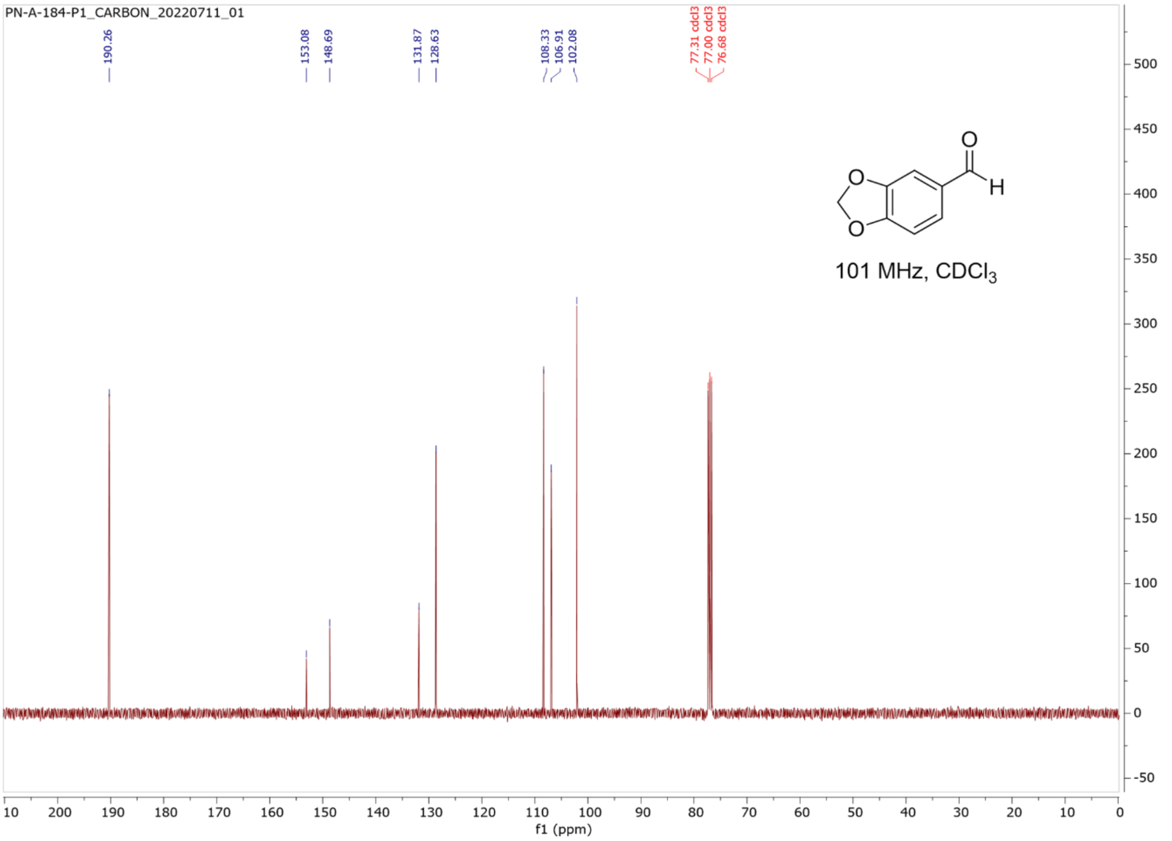

2-Iodobenzaldehyde **(2e):**

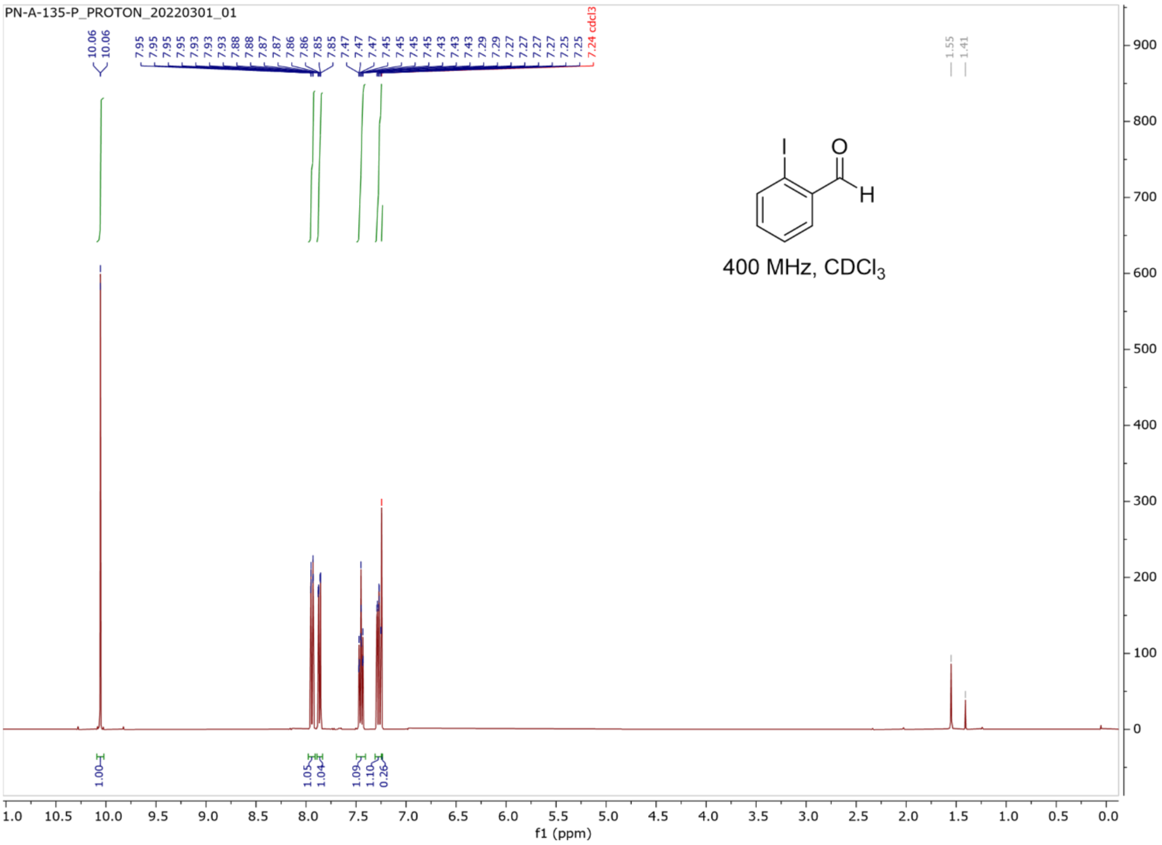

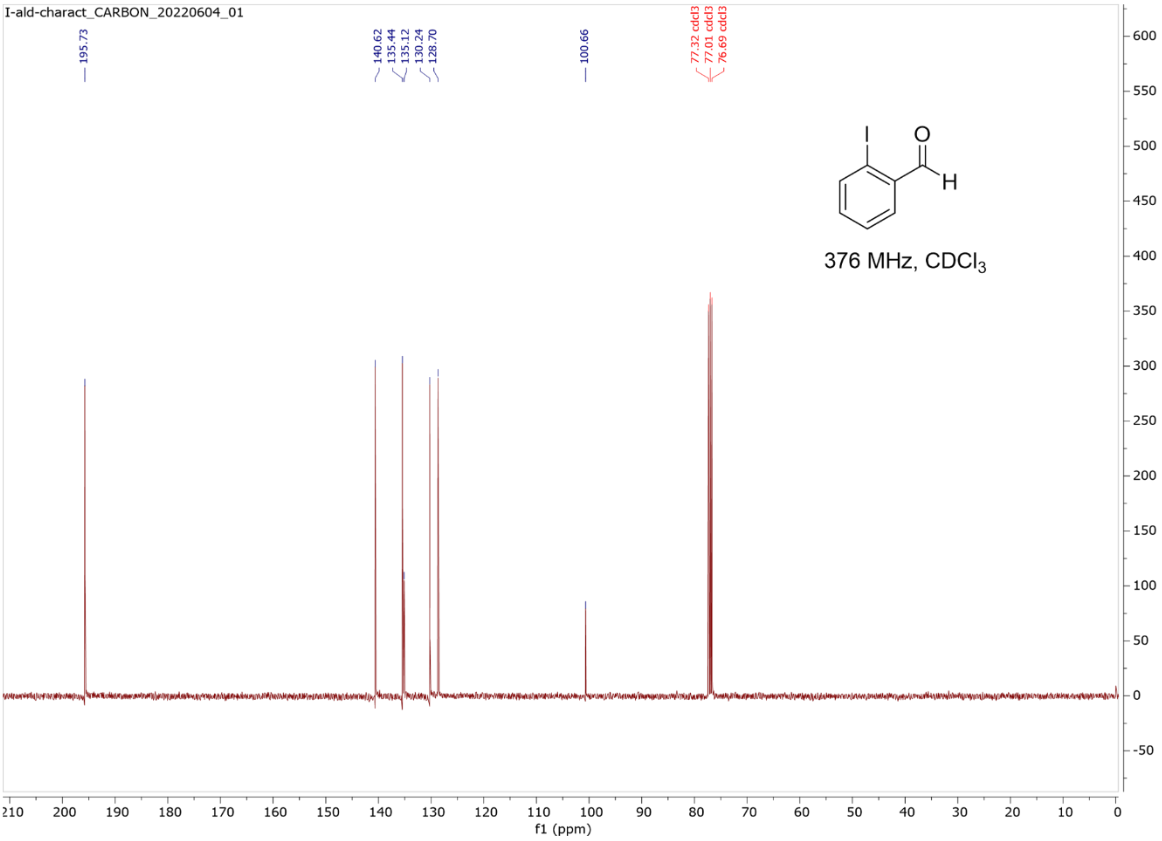

trans-Cinnamylaldehyde **(2f):**

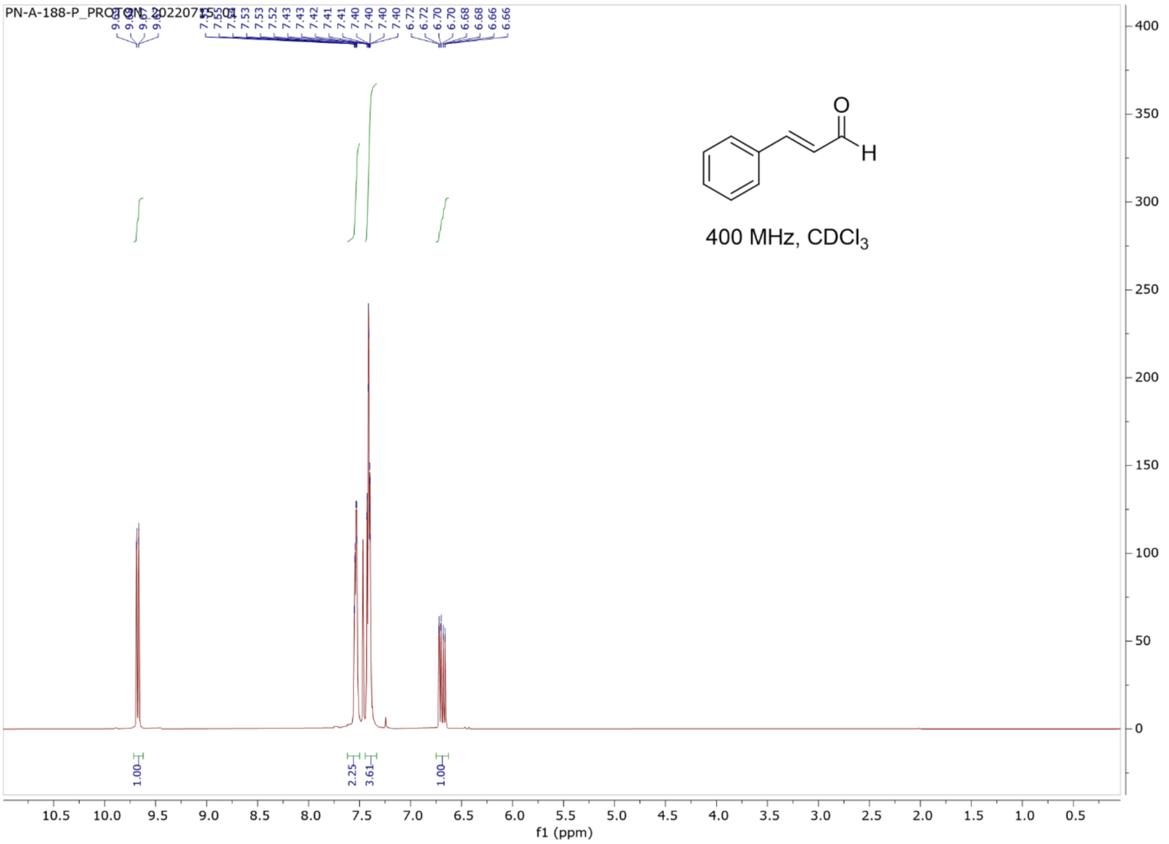

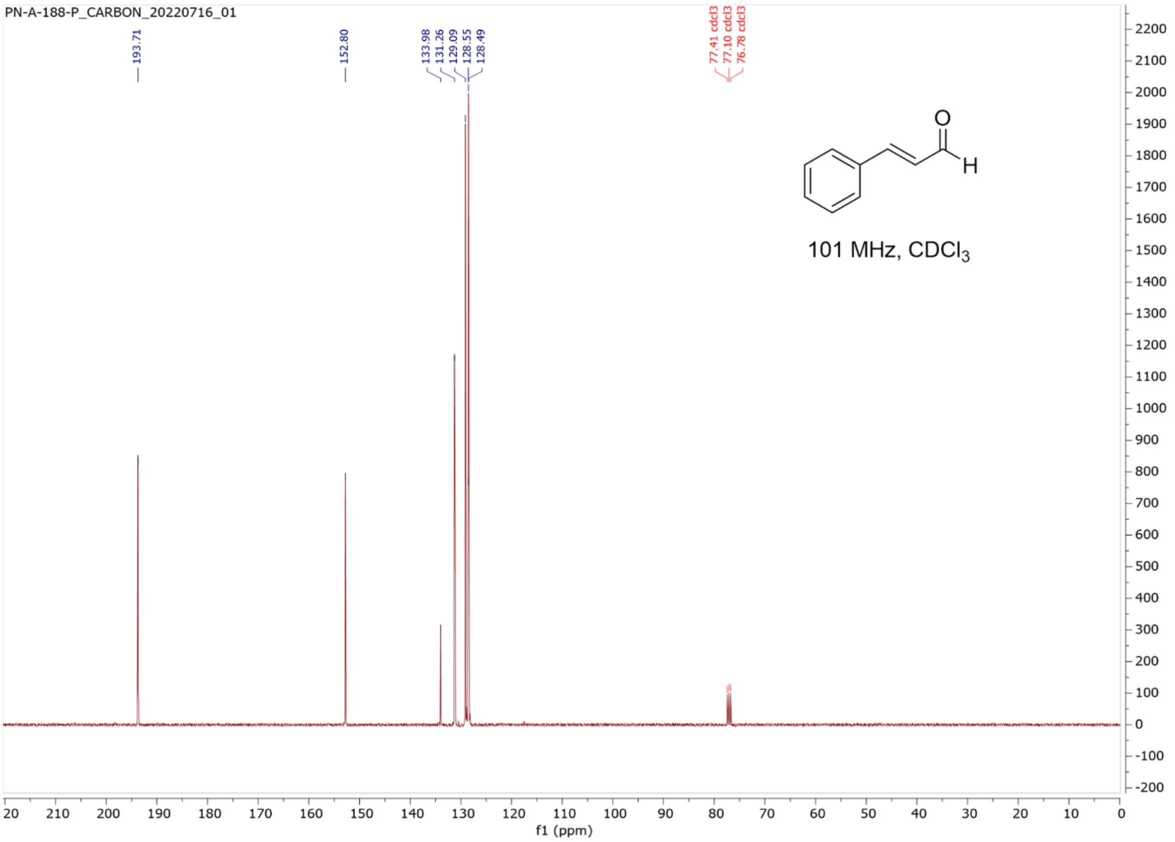

5-(Furan-2-yl)isoxazole-3-carbaldehyde **(2g):**

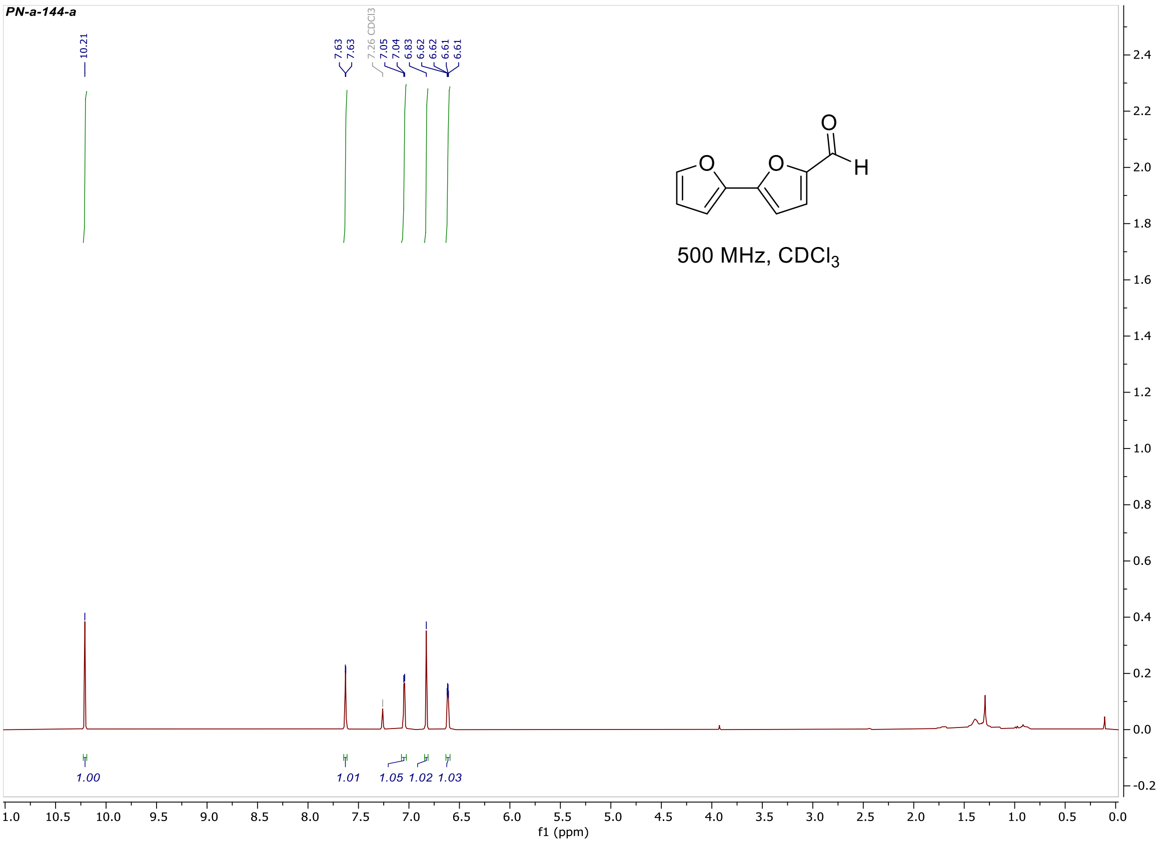

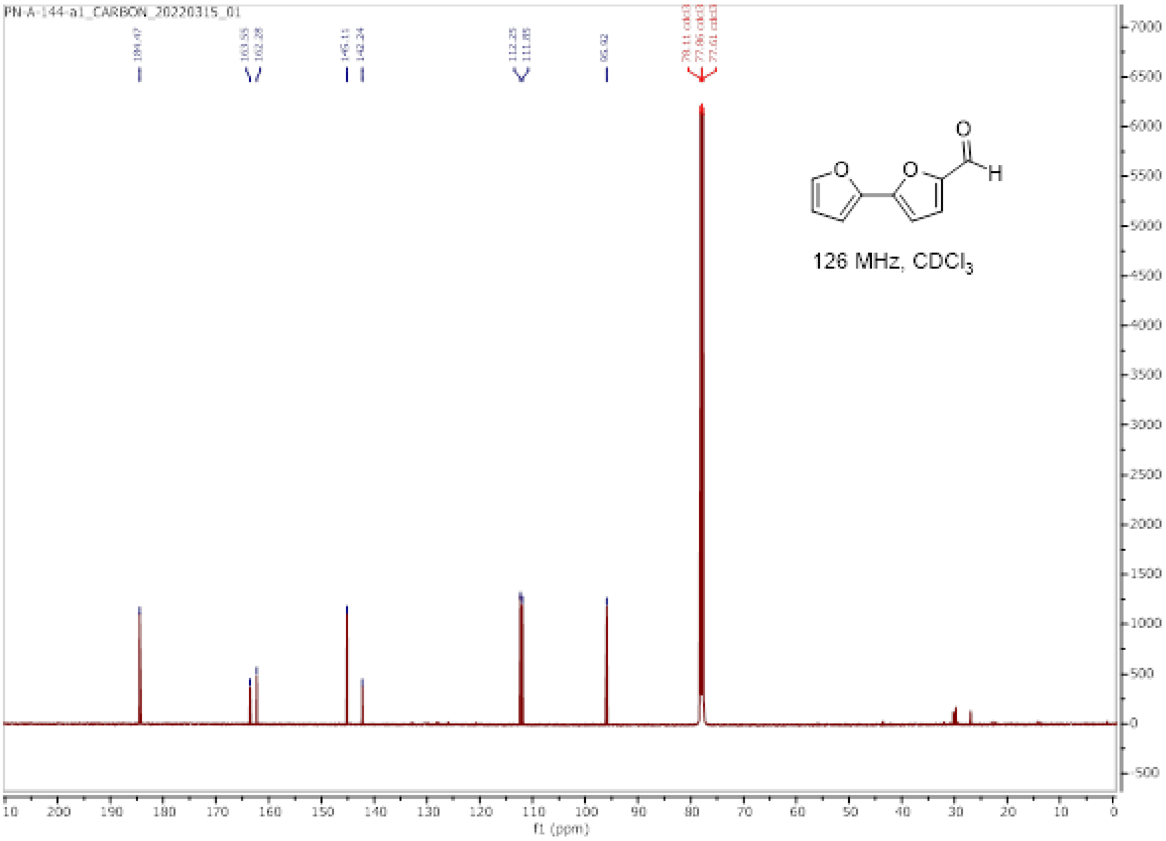

5-(4-Fluoro-3-(trifluoromethyl)phenyl)isoxazole-3-carbaldehyde **(2h):**

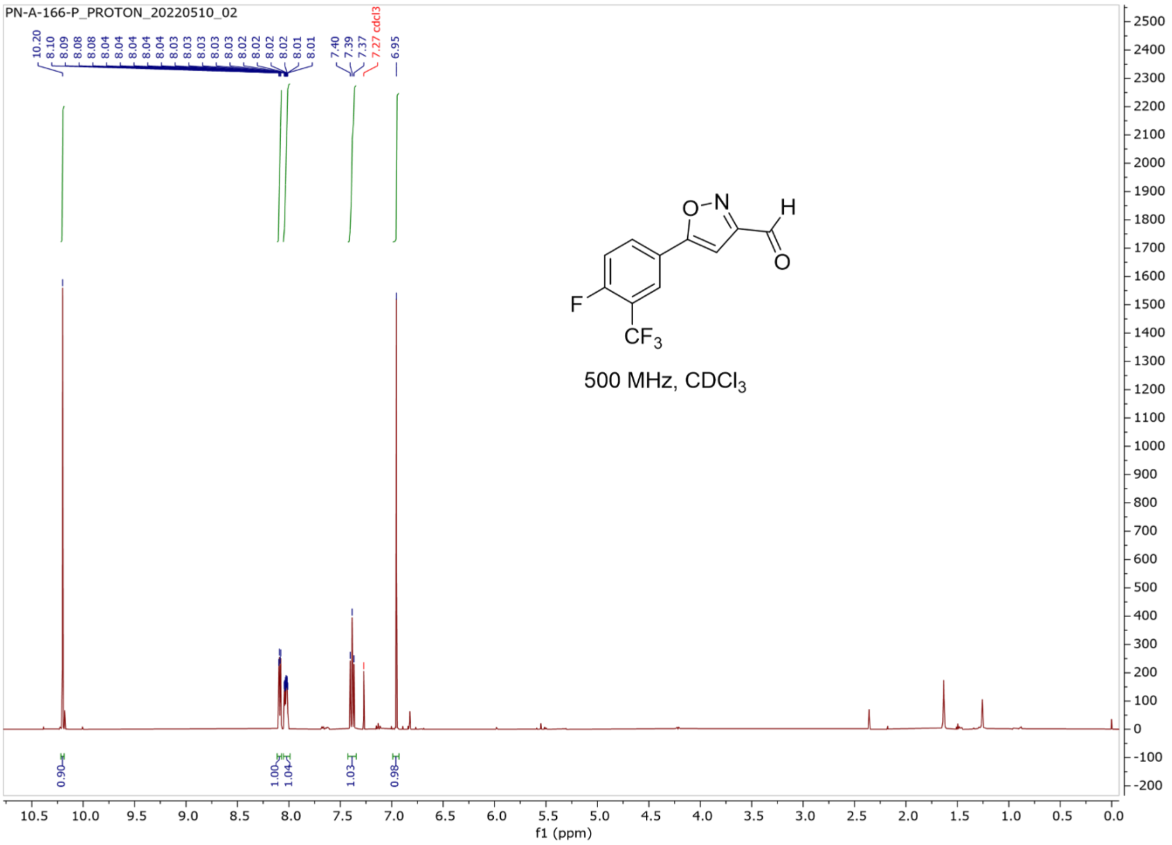

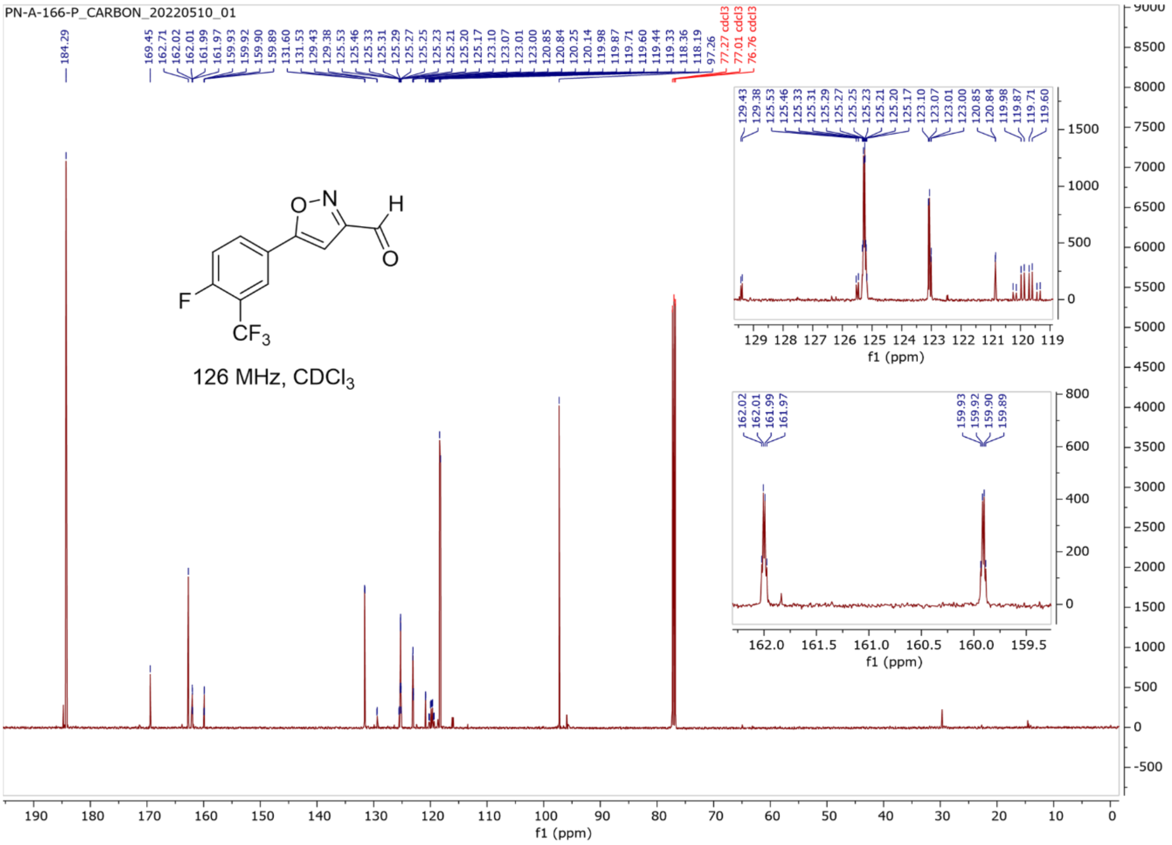

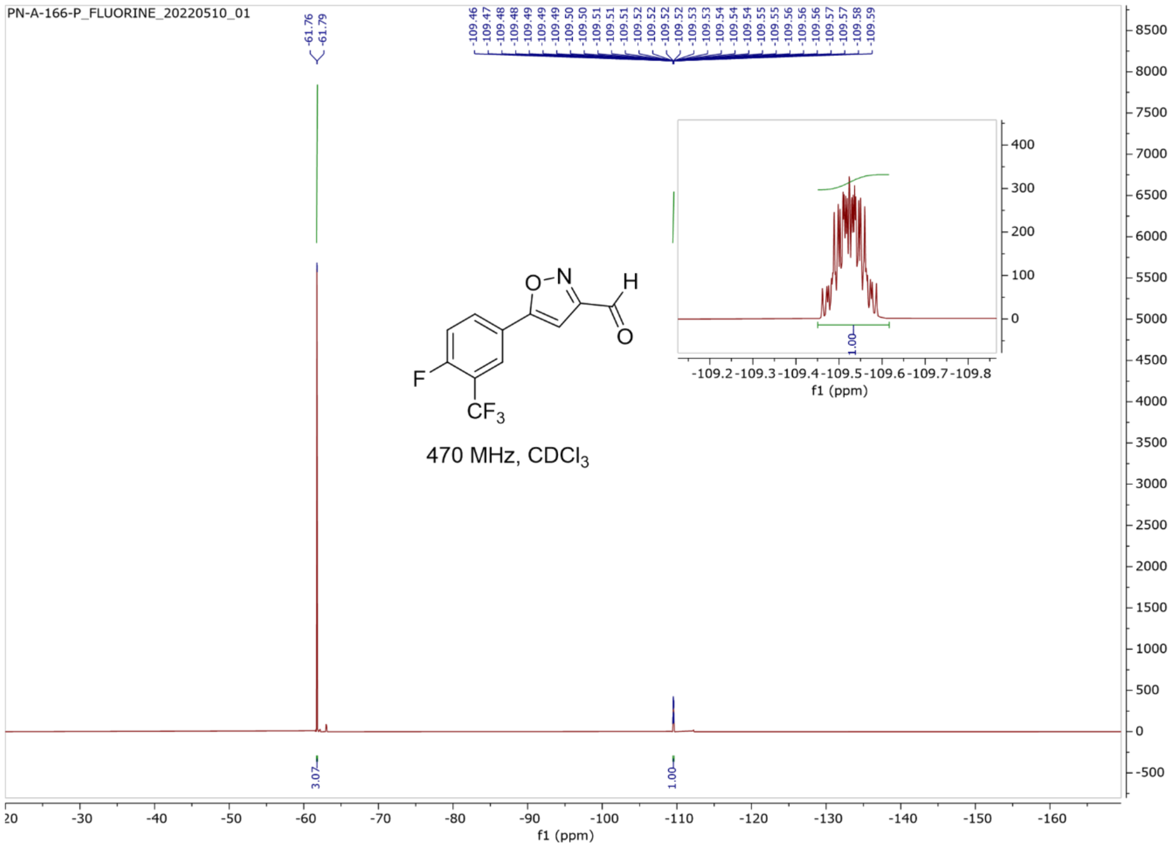
PN-A-166-P_FLUORINE_20220510_01
-61.76
-61.79
470 MHz, CDCl3
3.07
1.00
f1 (ppm)

5-(2,4-Dichloro-5-fluorophenyl)isoxazole-3-carbaldehyde **(2i):**

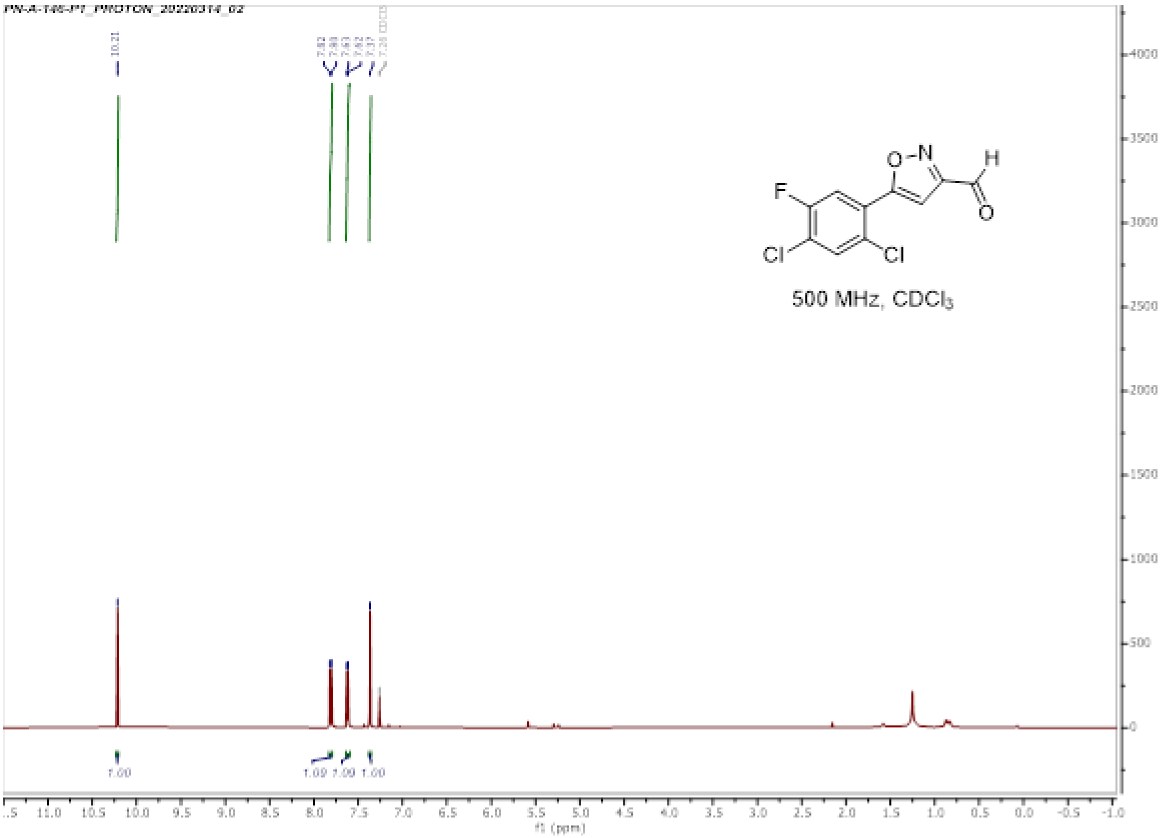

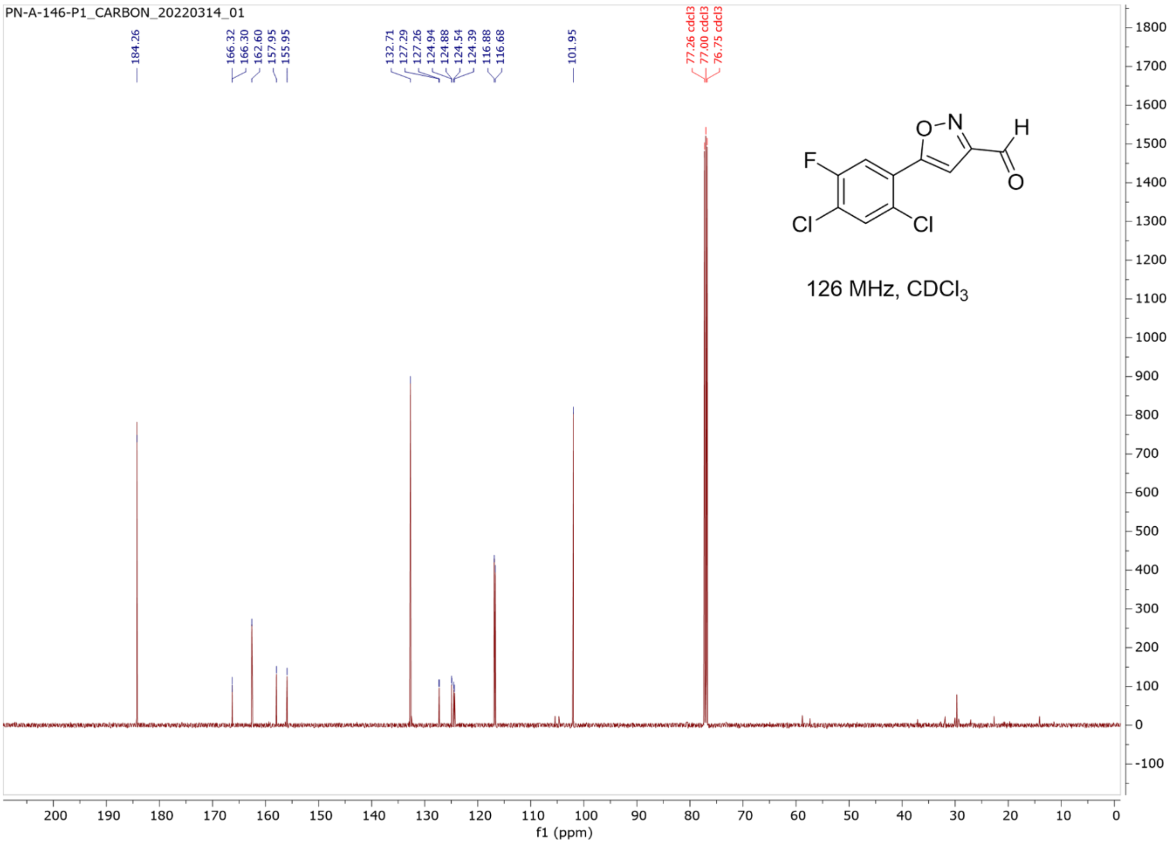

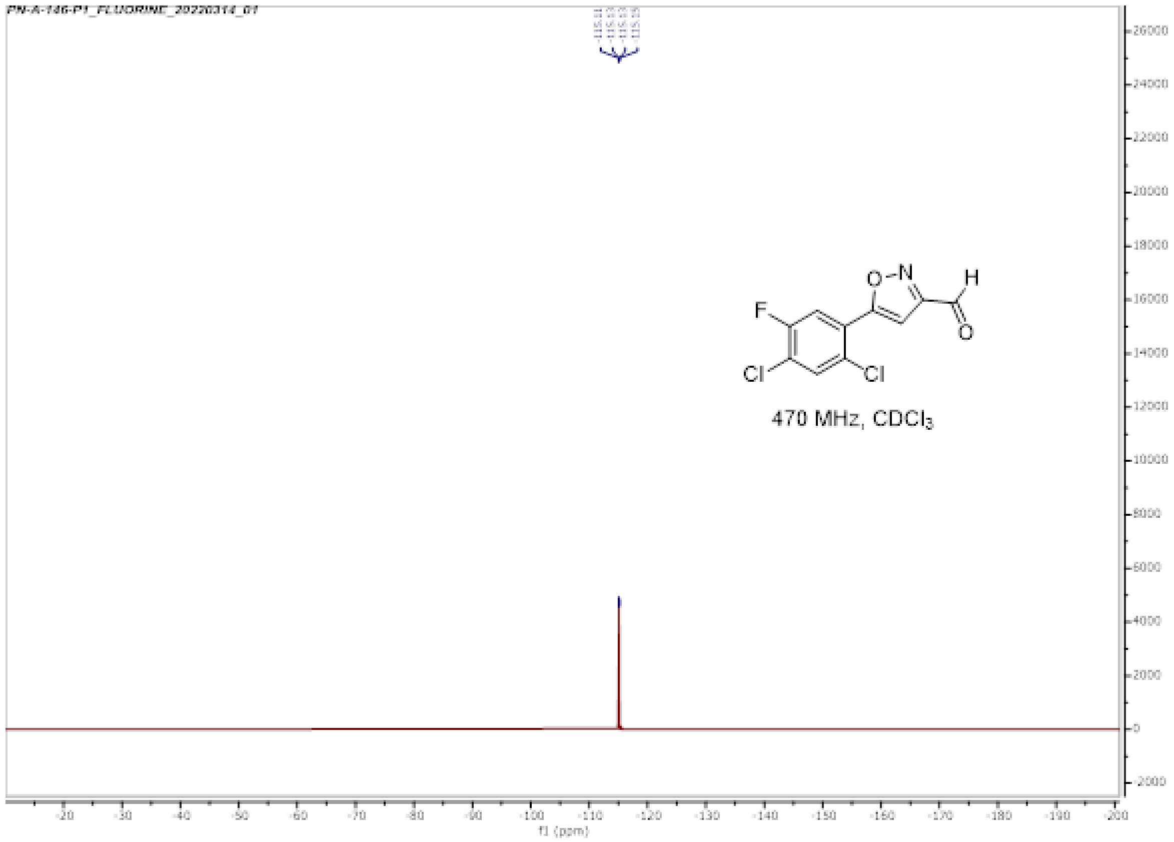
PN-A-146-P1_FLUORINE_20220314_01
470 MHz, CDCl3
f1 (ppm)

Benzophenone **(2j):**

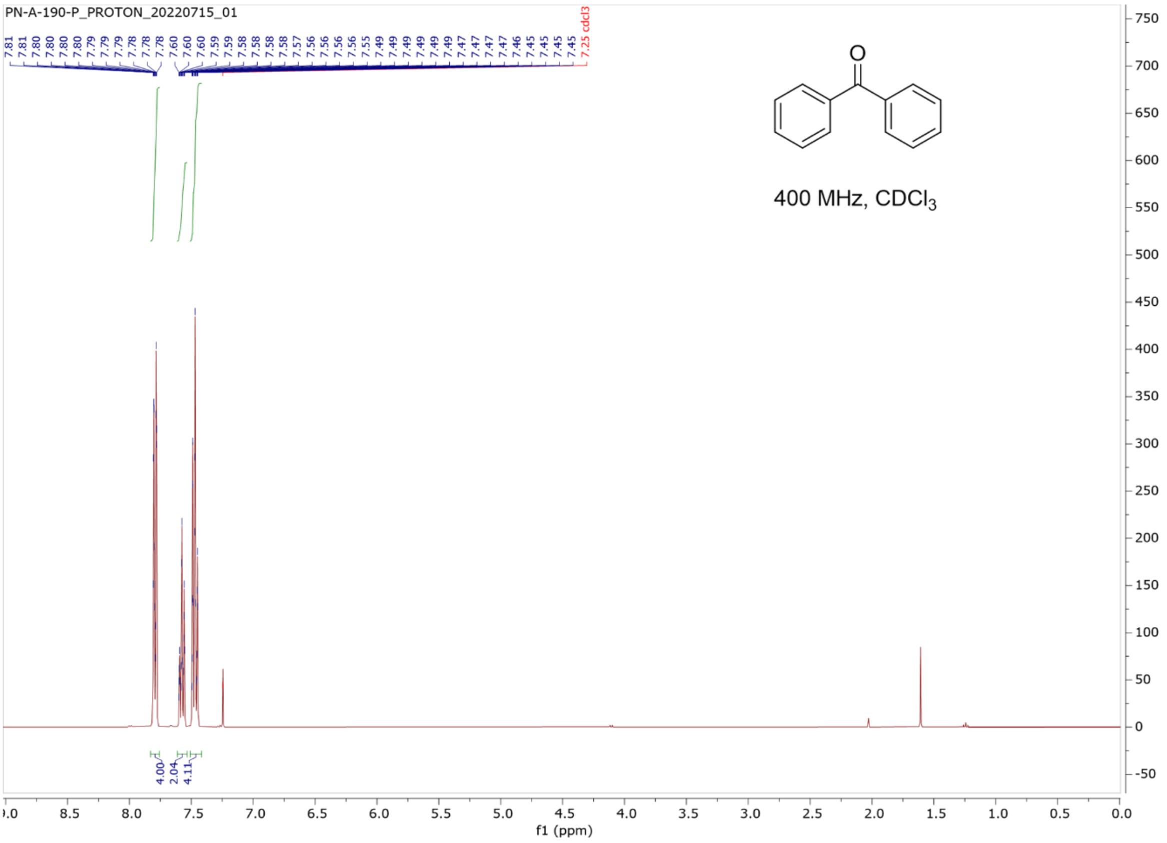

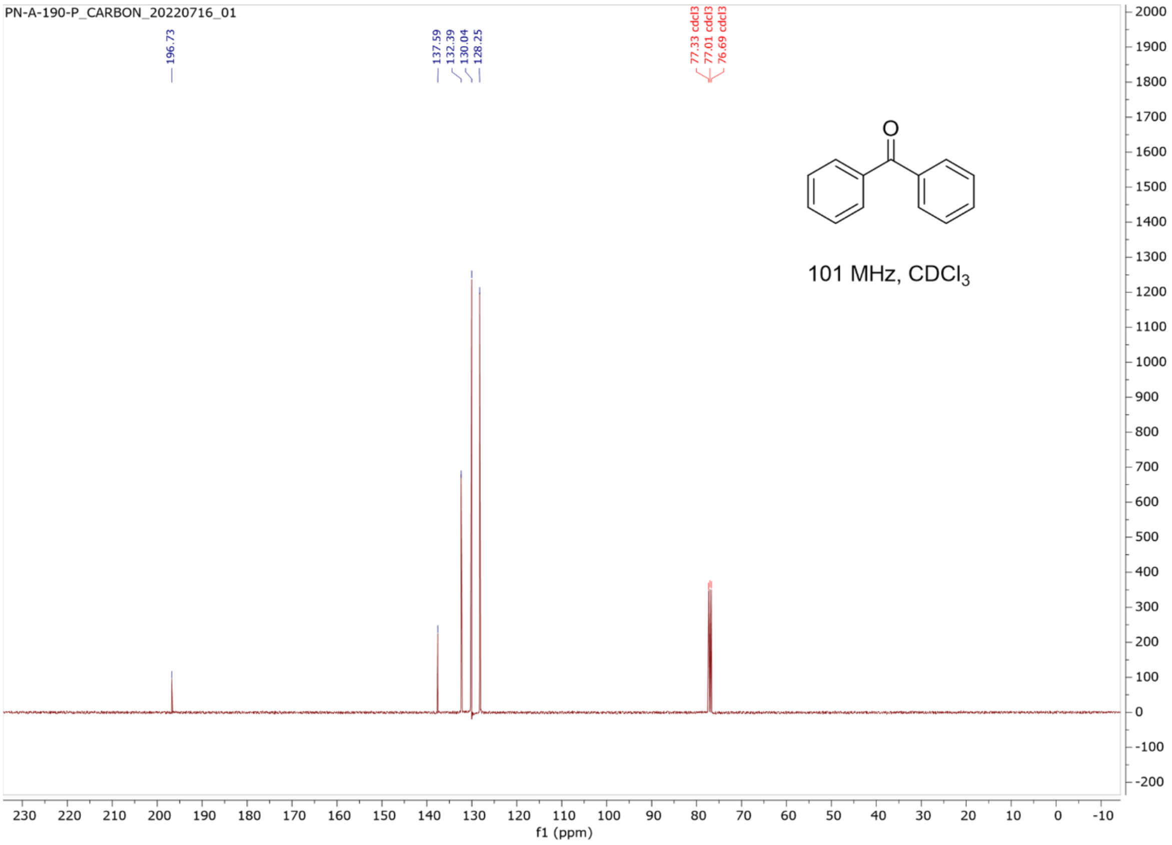

1-(2,4-Difluorophenyl)-3-(pyridin-2-yl)propan-1-one **(2k)**

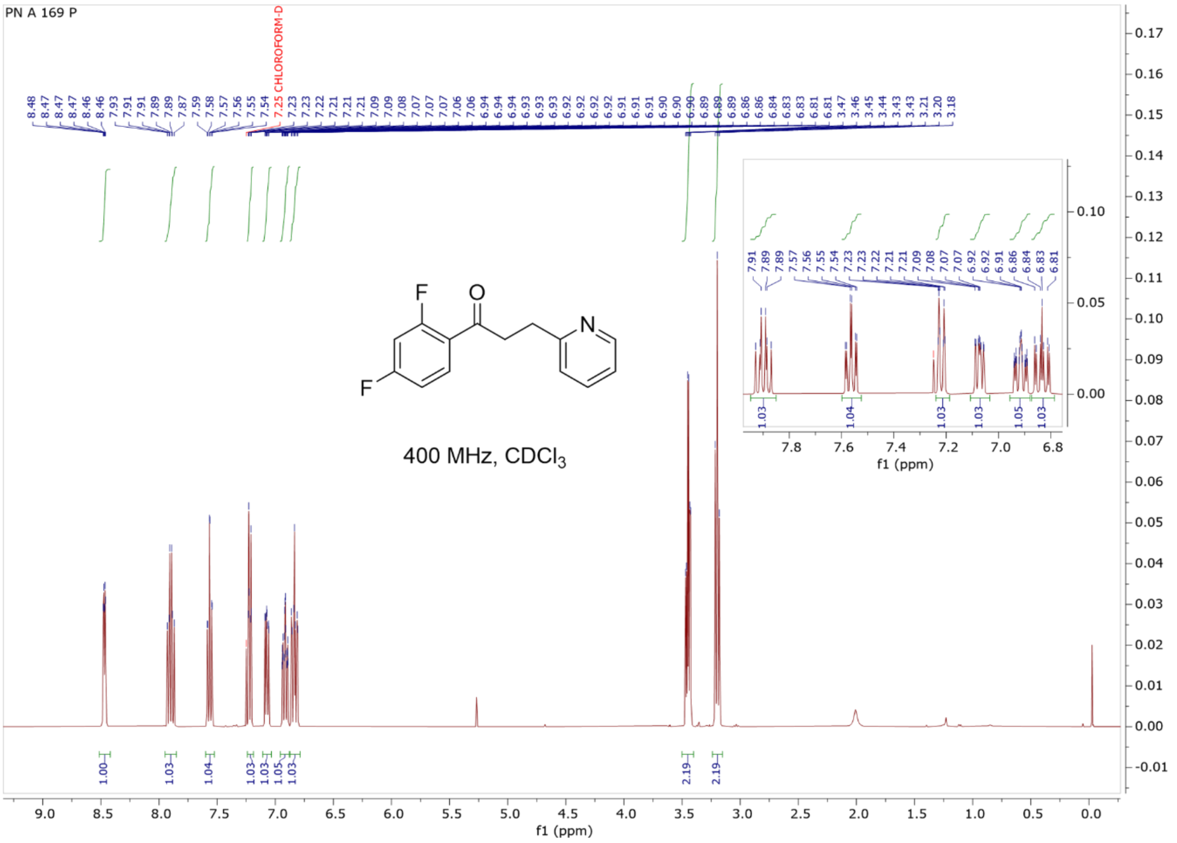

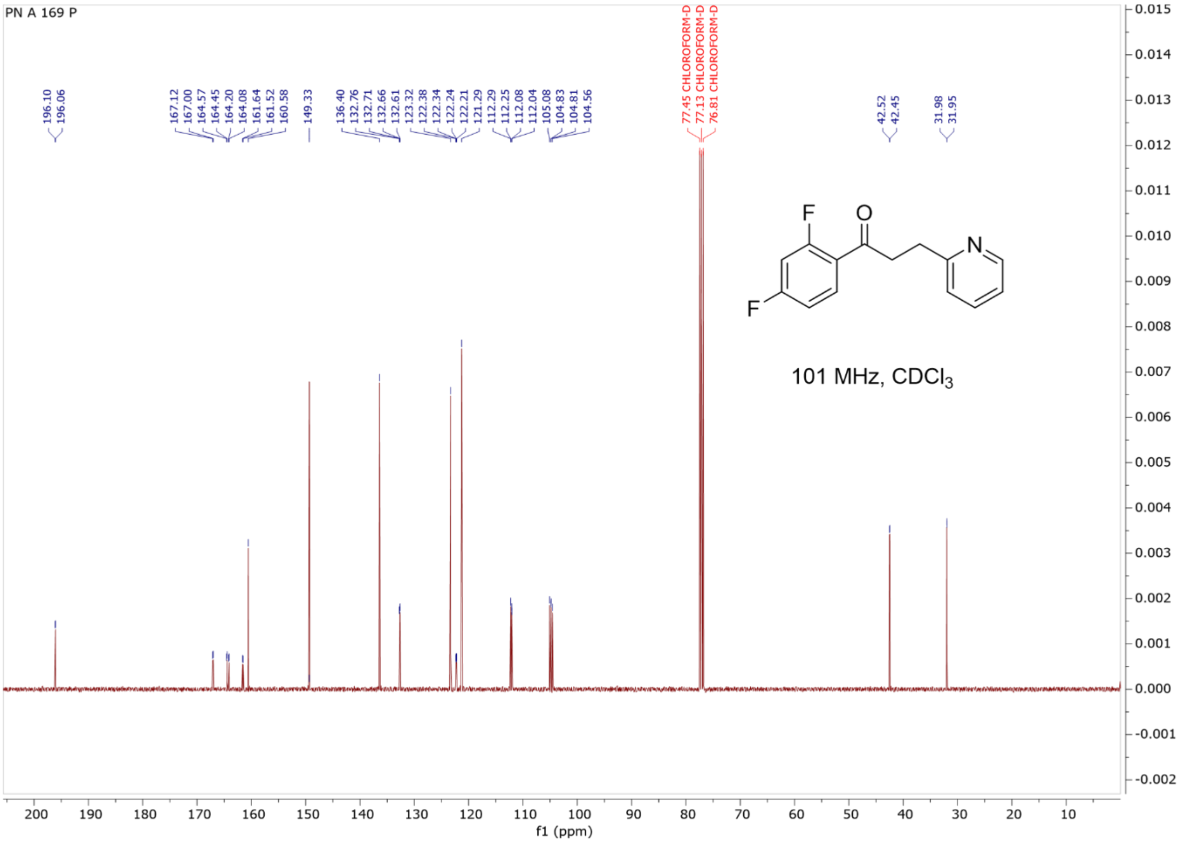

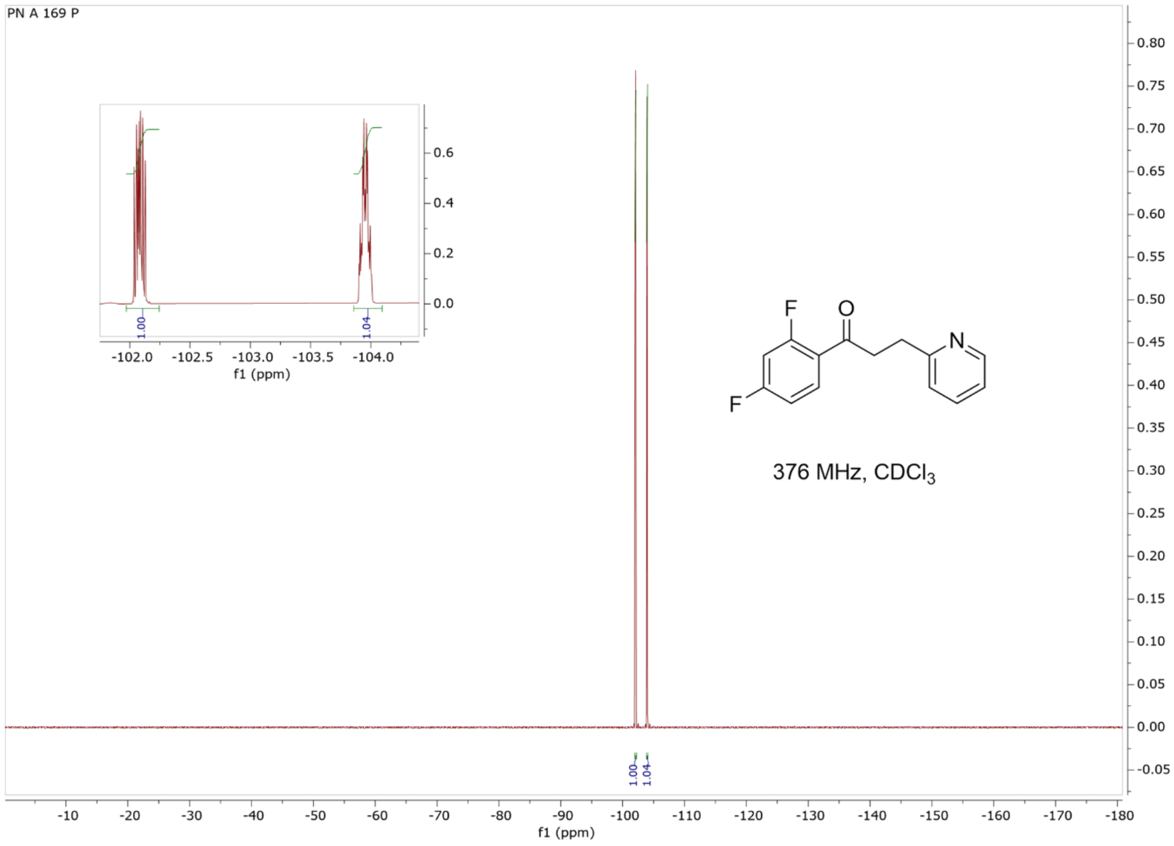
PN A 169 P
376 MHz, CDCl3
f1 (ppm)

1-(Thiazol-2-yl)ethan-1-one) **(2l):**

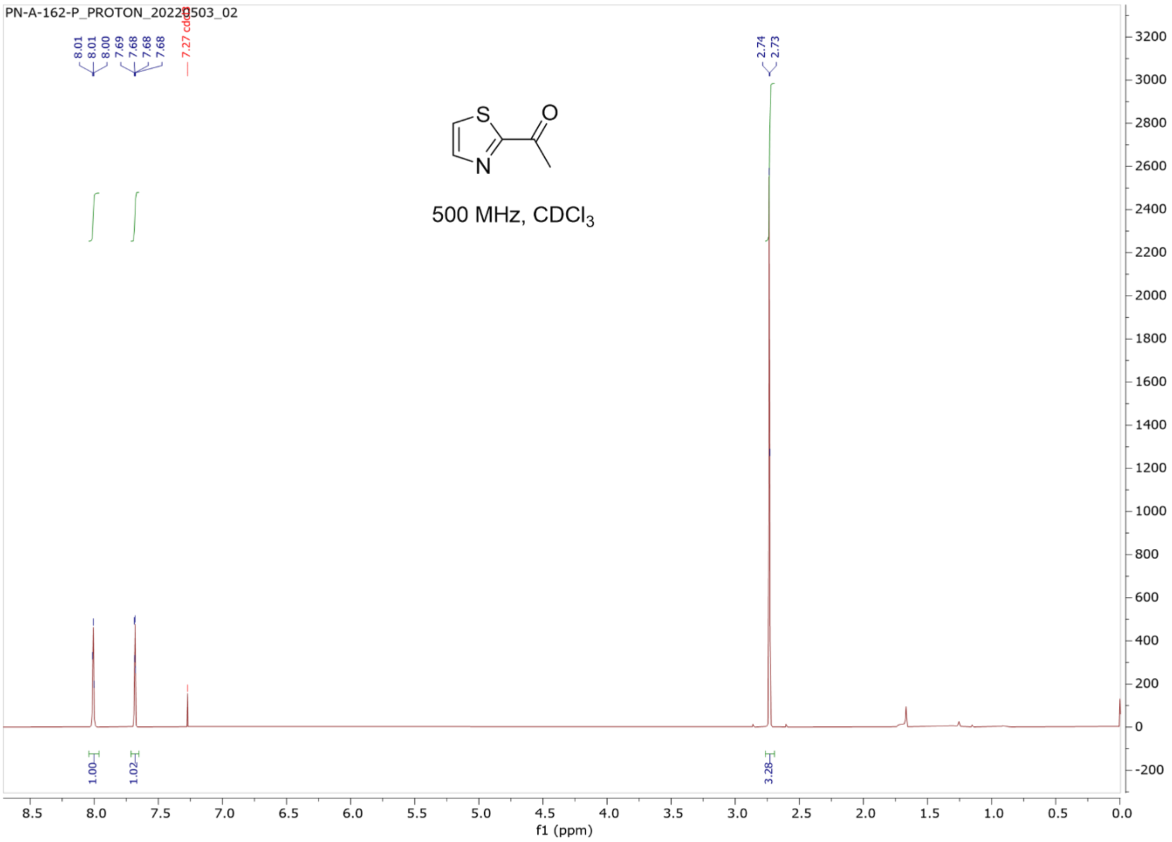

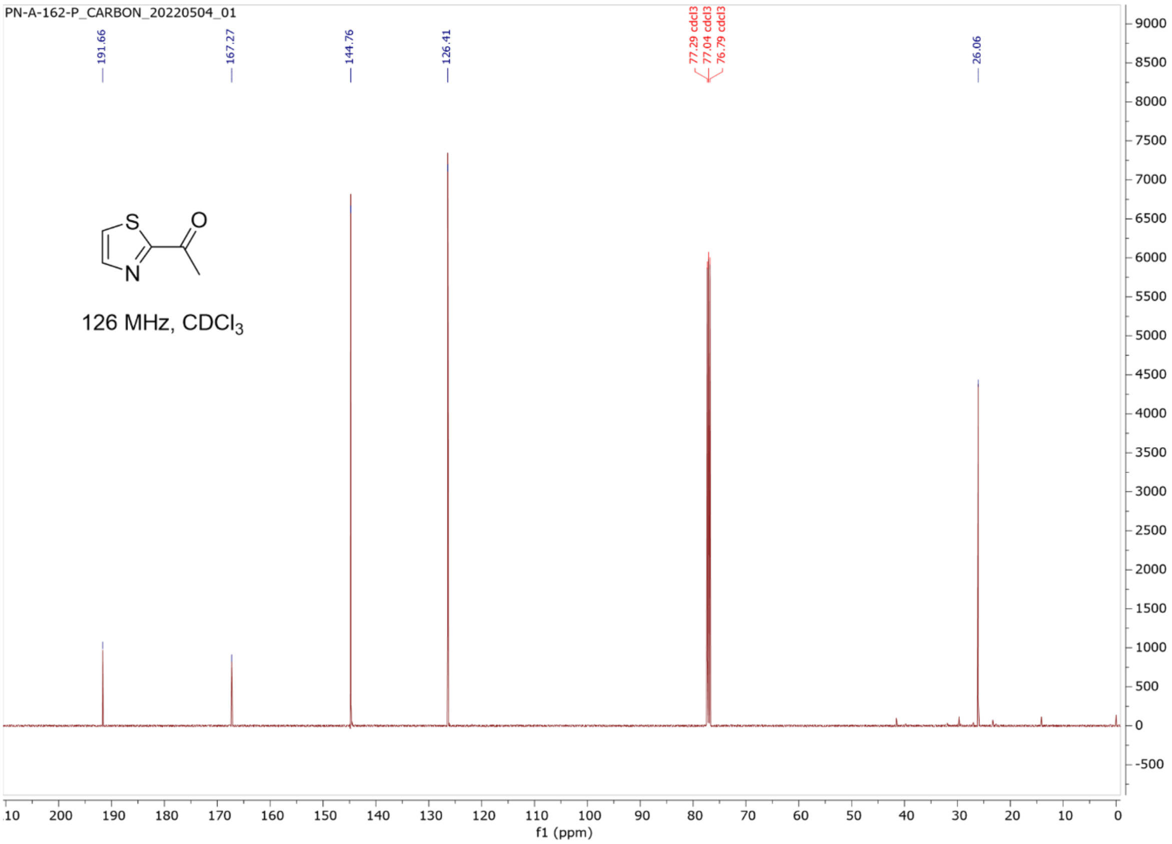

Cyclopentanone **(2m):**

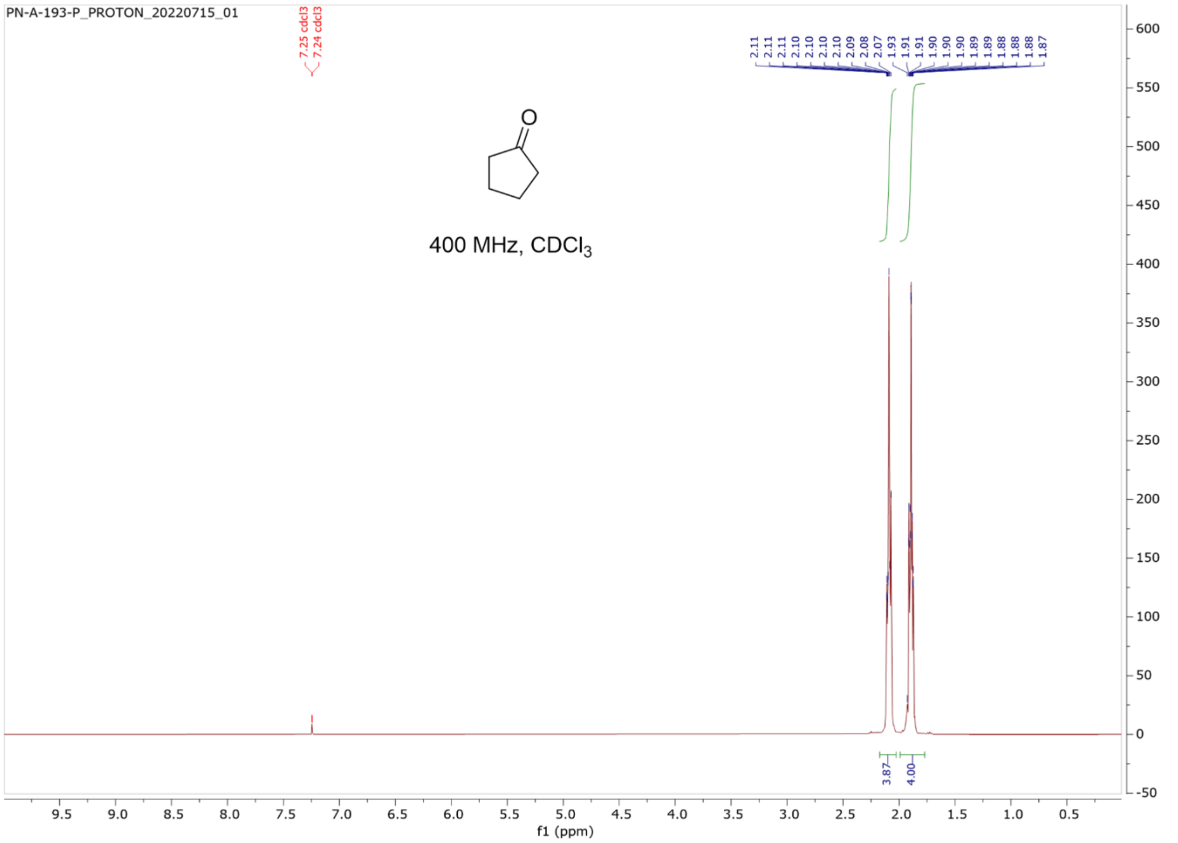

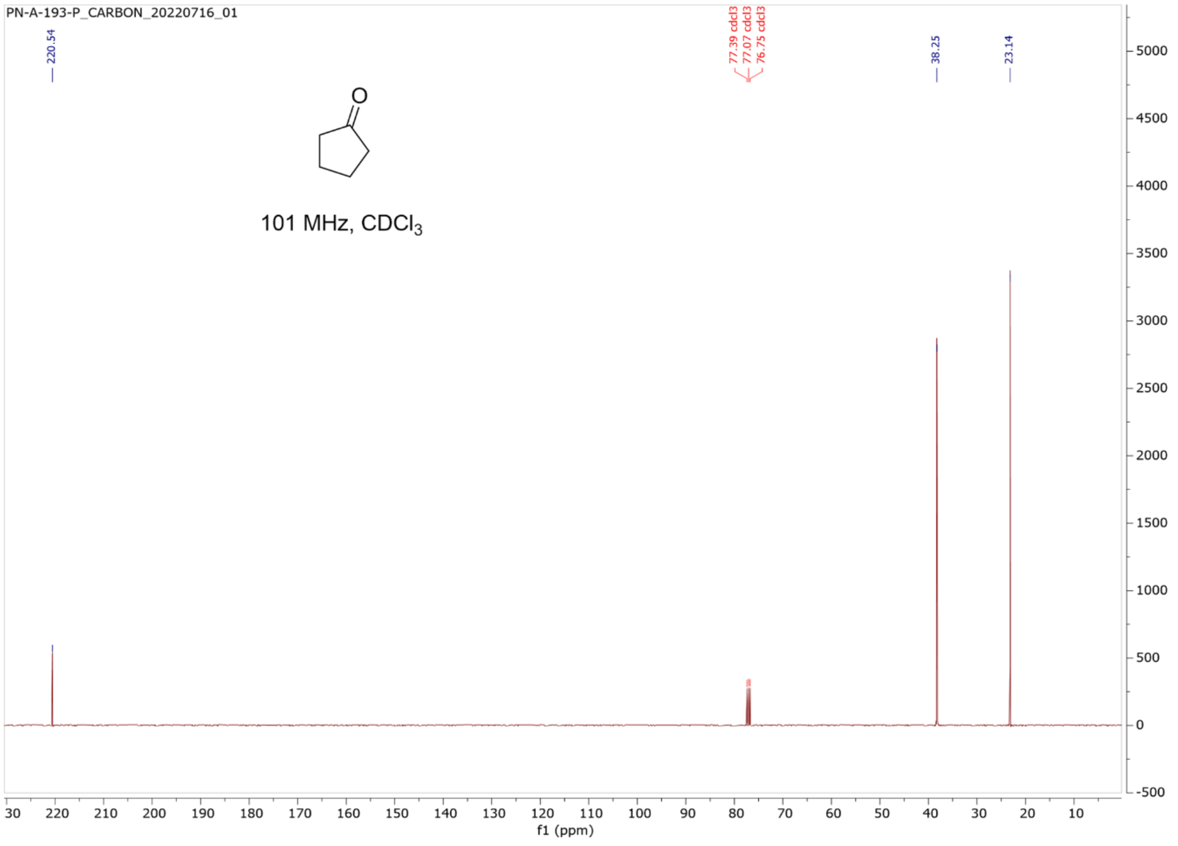

3,7-Dimethyloctanal **(2o):**

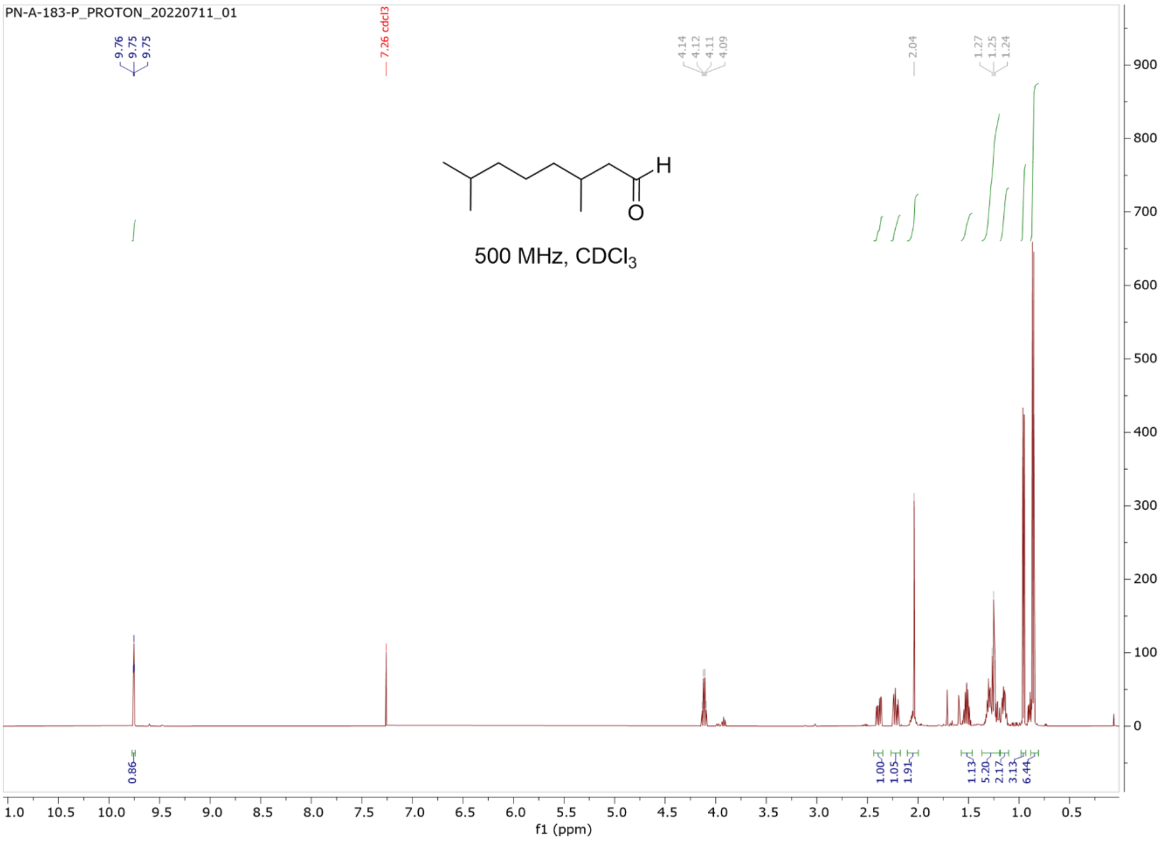

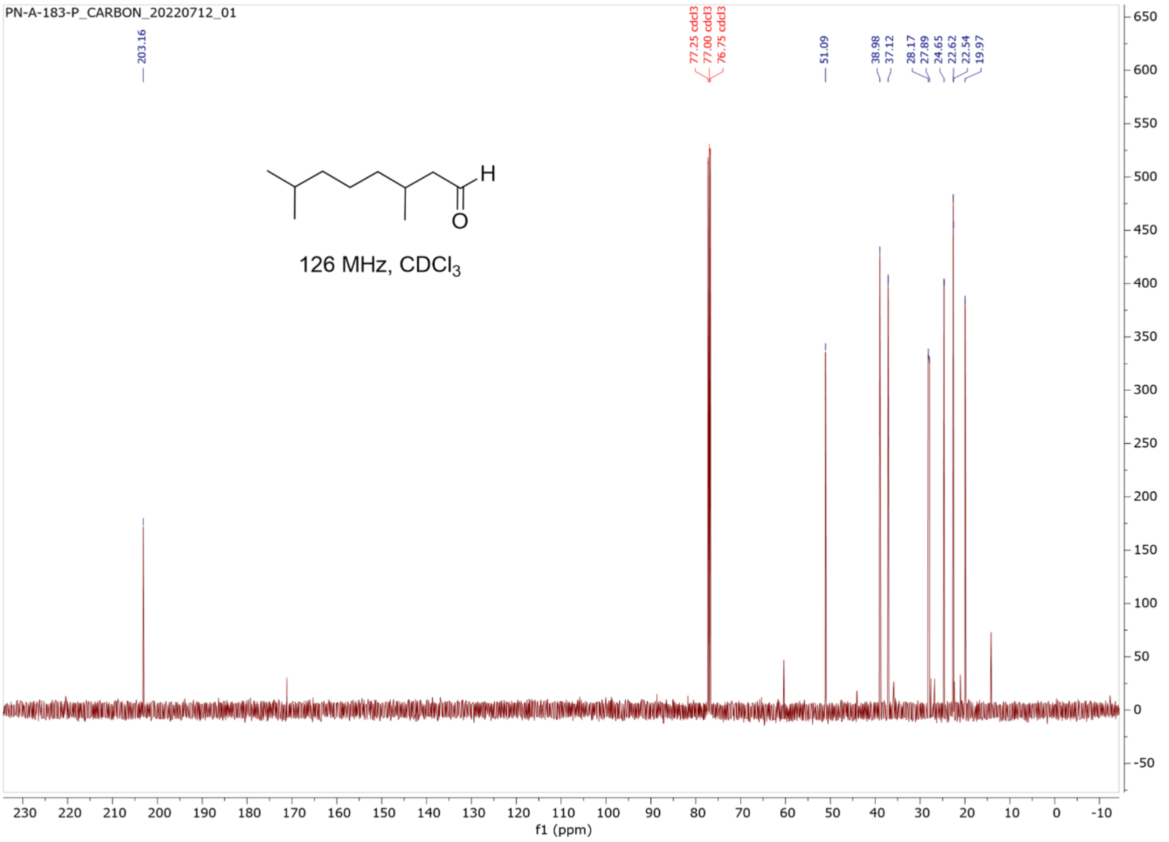

(rac)-4,4-Difluoro-*N*-(3-oxo-1-phenylpropyl)cyclohexane-1-carboxamide **(2u):**

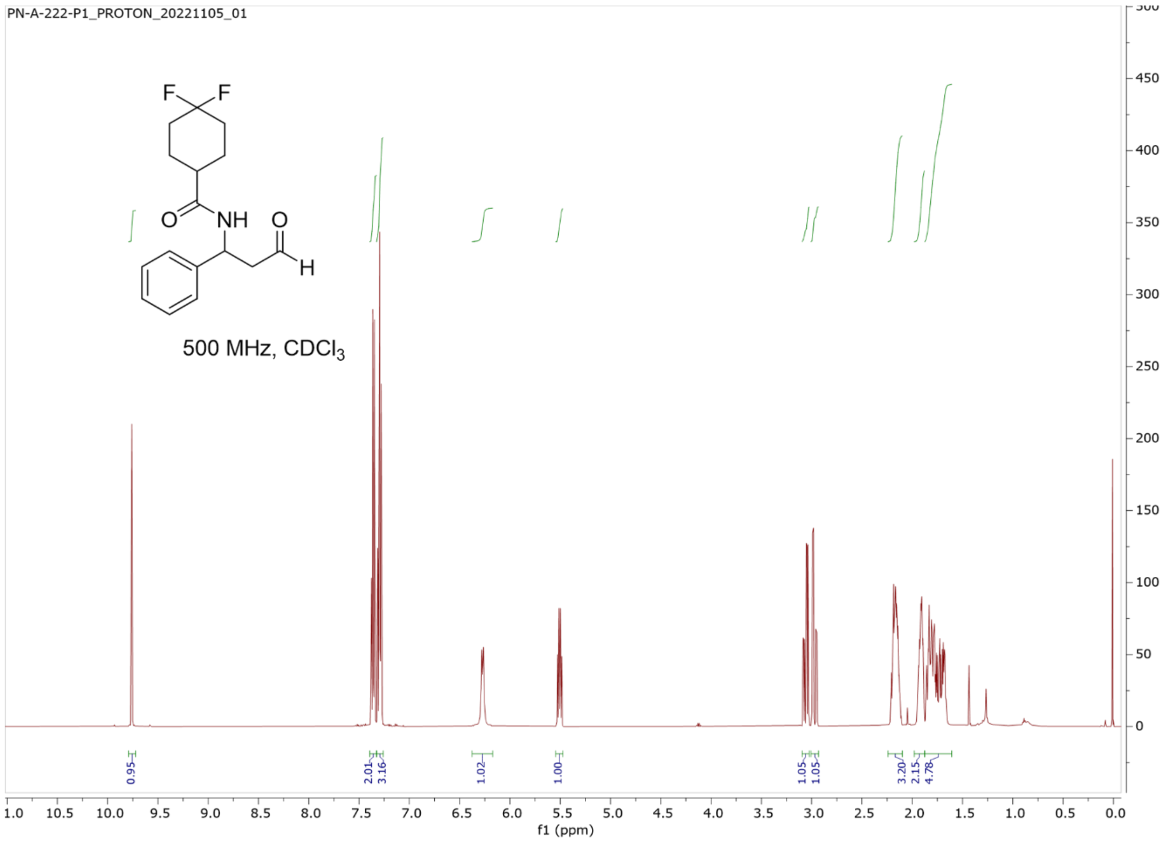

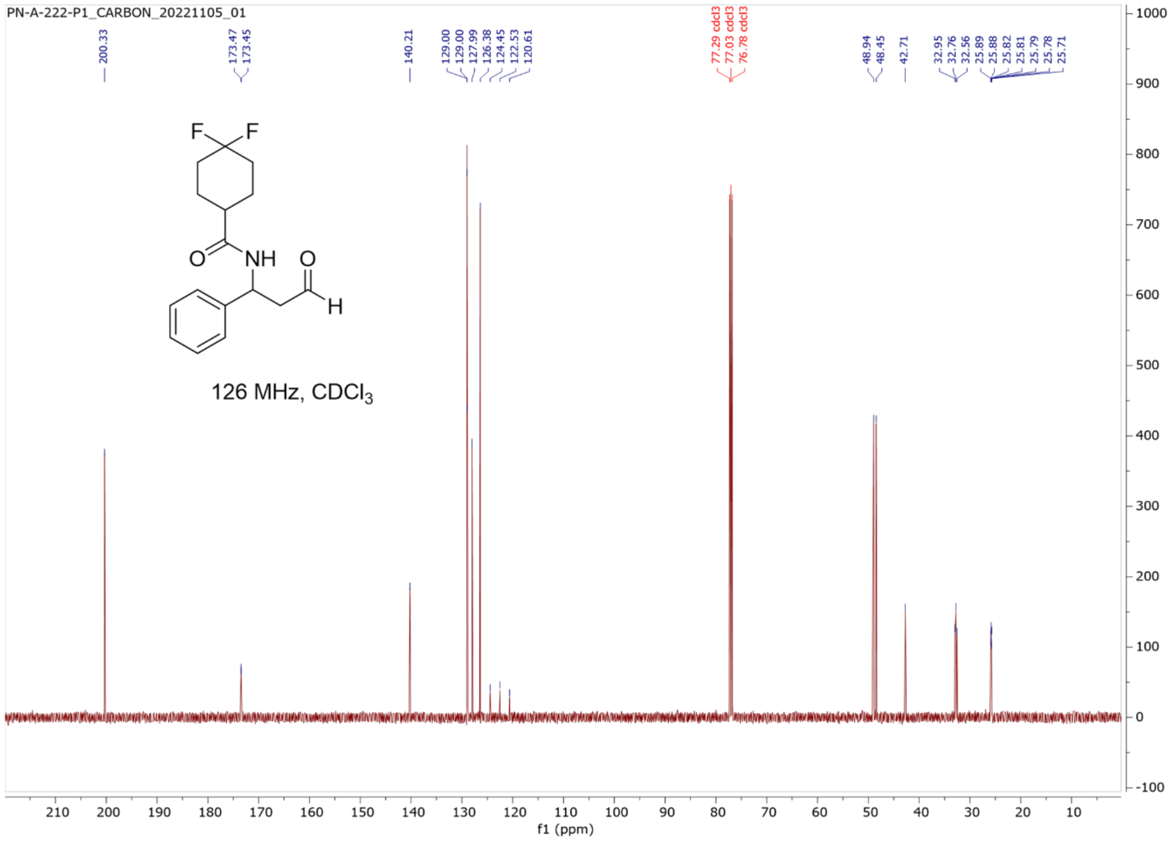

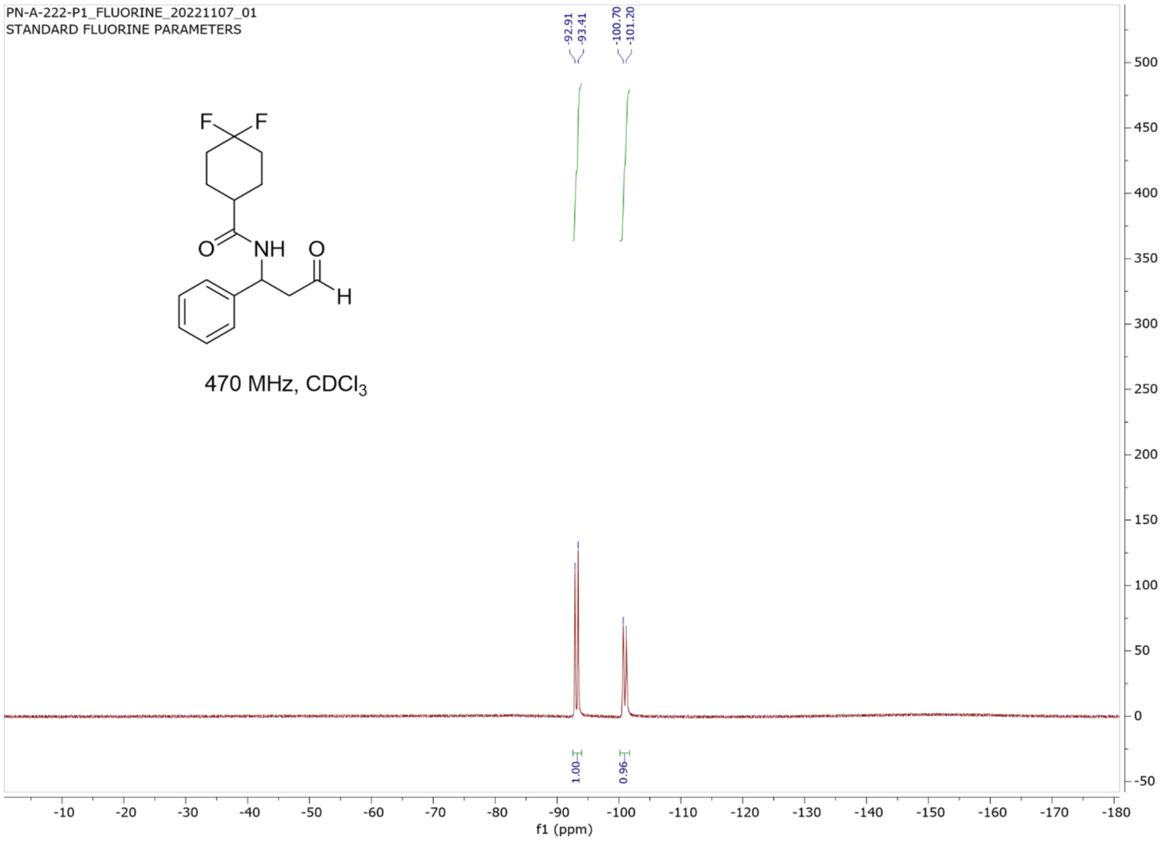

PN-A-222-P1_FLUORINE_20221107_01
STANDARD FLUORINE PARAMETERS
470 MHz, CDCl3
-92.91
-93.41
-100.70
-101.20
1.00
0.96
f1 (ppm)

3-(2-Phenyl-5-(trifluoromethyl)oxazol-4-yl)propan-1-ol **(1b):**

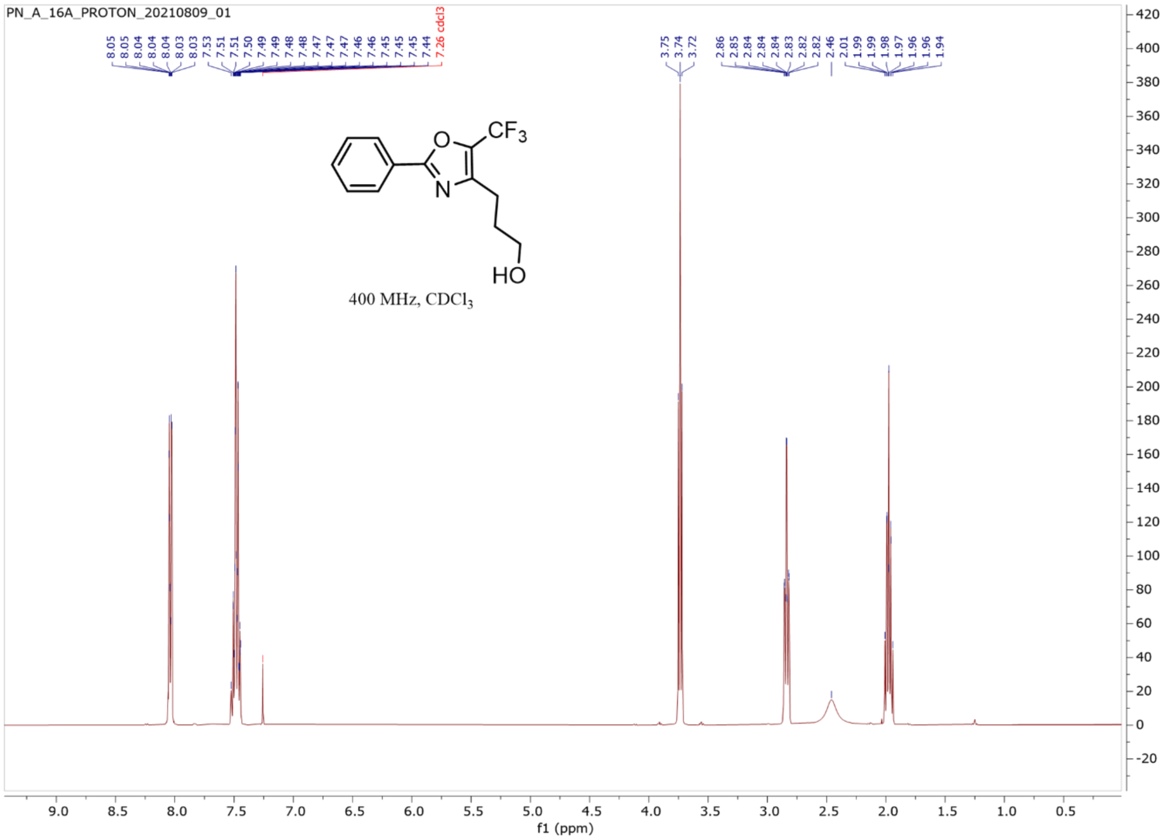

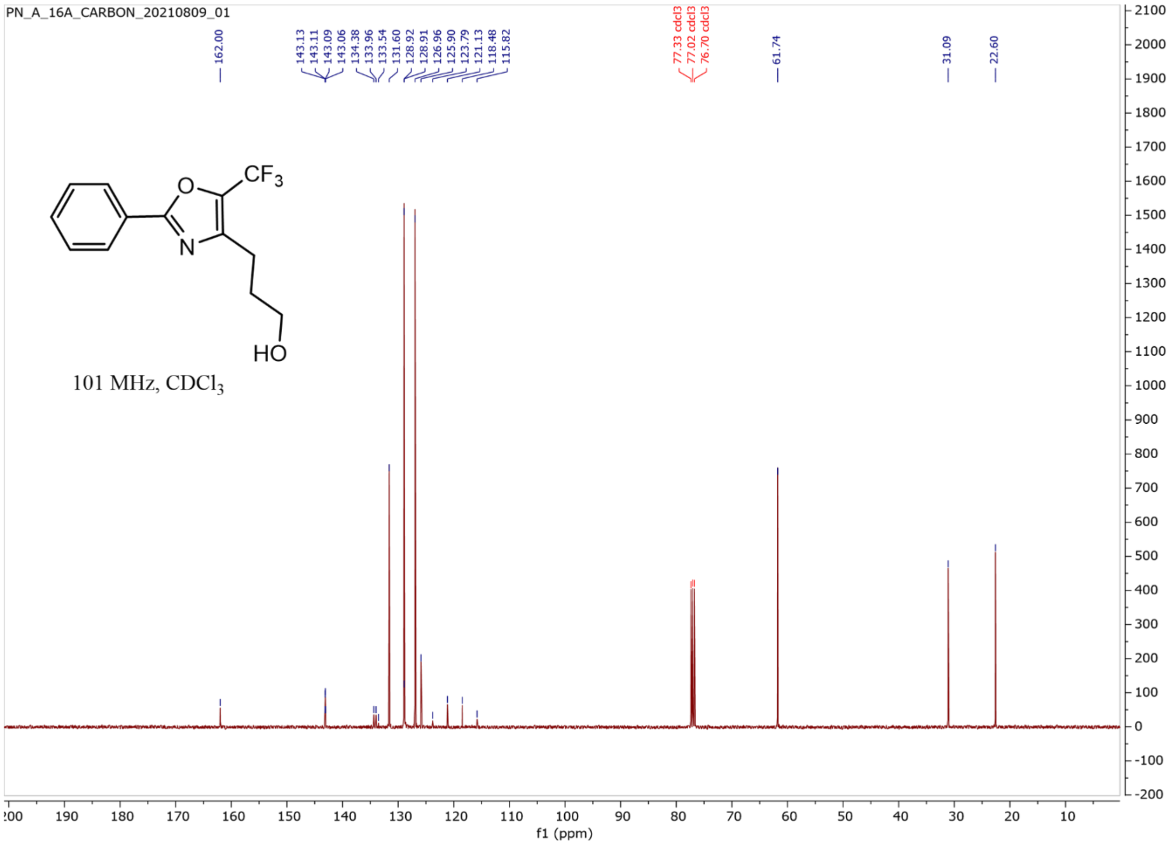

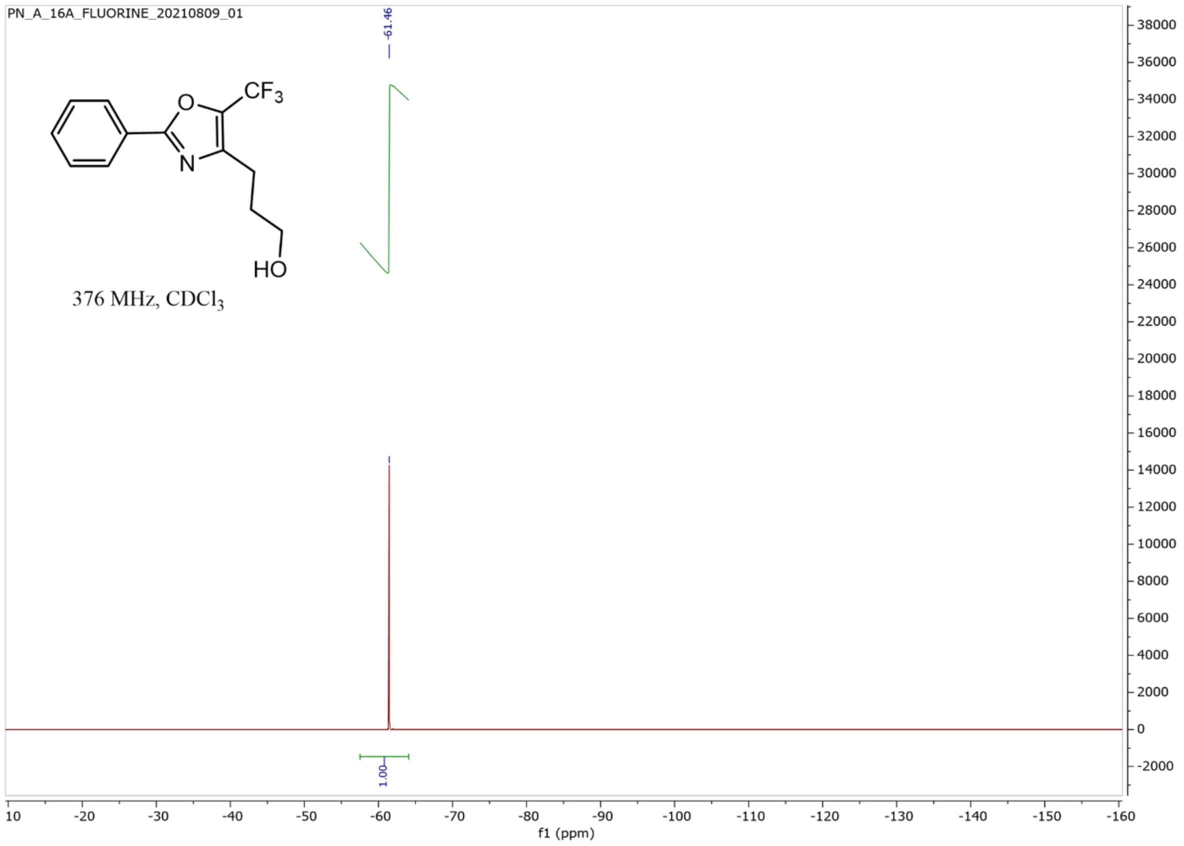
PN_A_16A_FLUORINE_20210809_01
-61.46
CF3
O
N
HO
376 MHz, CDCl3
1.00
f1 (ppm)

3-(2-Phenyl-5-(trifluoromethyl)oxazol-4-yl)propyl 2,2,2-trifluoroacetate (**5**):

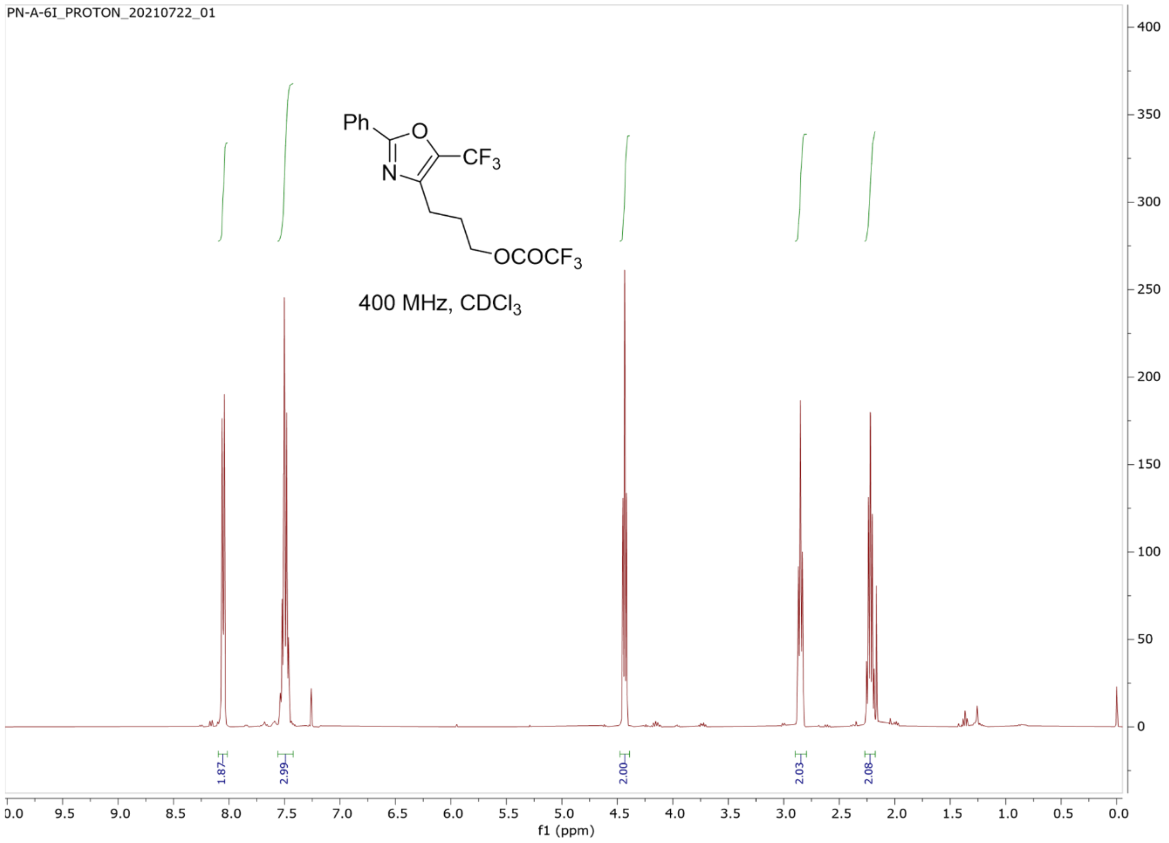

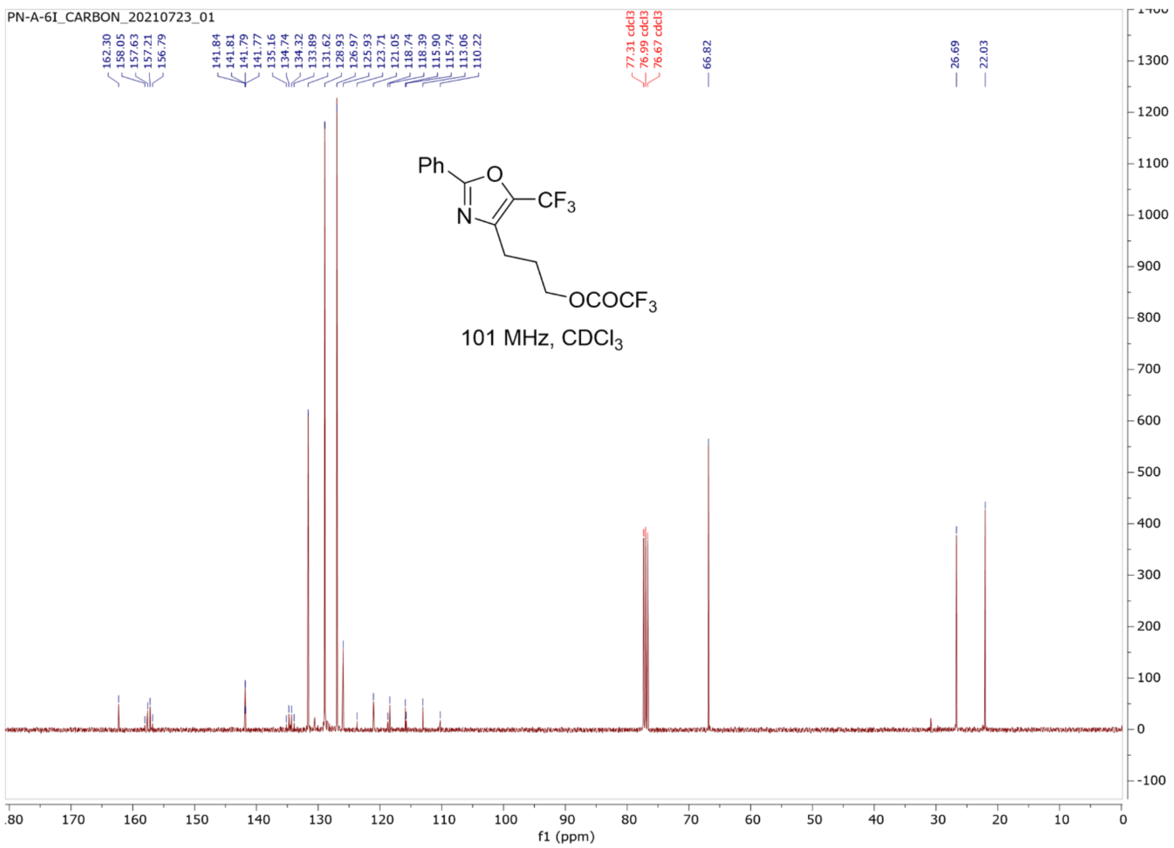

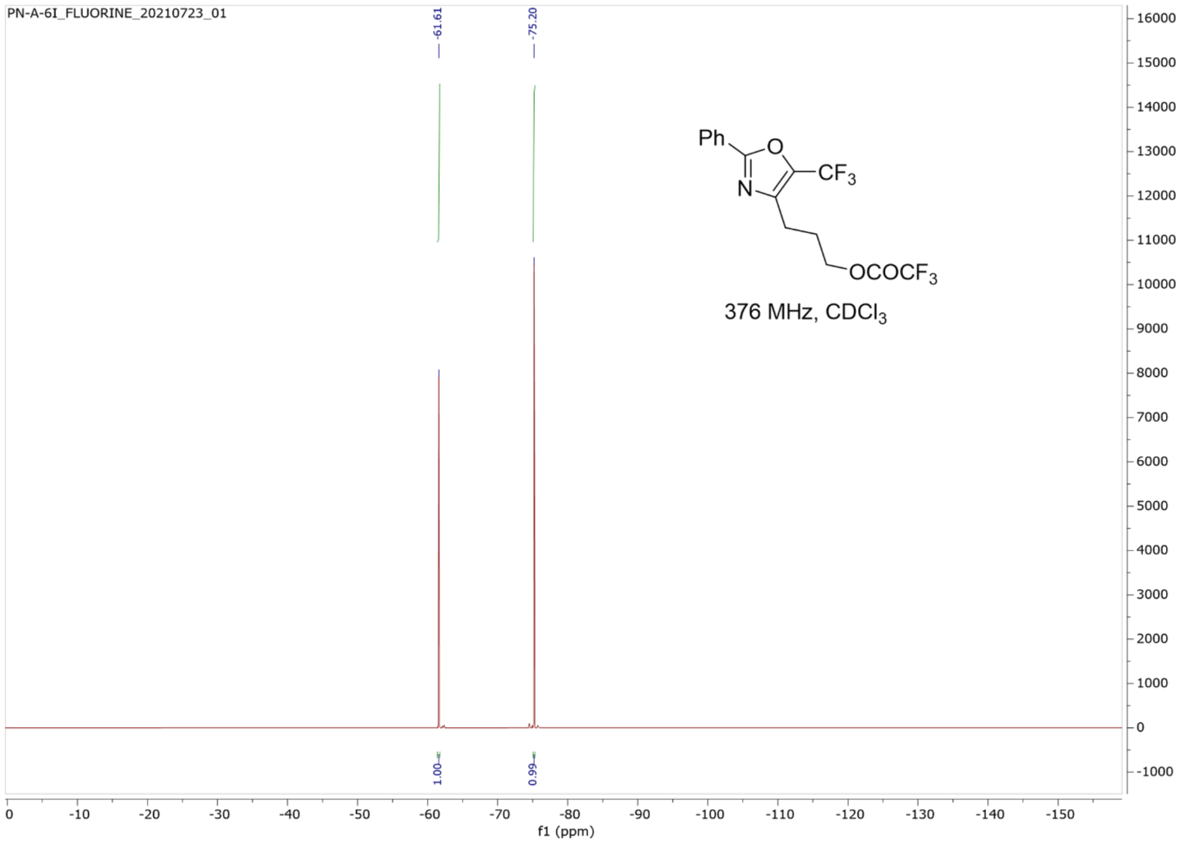
PN-A-6I_FLUORINE_20210723_01
-61.61
-75.20
Ph
O
N
CF3
OCOCF3
376 MHz, CDCl3
1.00
0.99
f1 (ppm)
